# High Strength, Strain, and Resilience of Gold Nanoparticle Reinforced Eutectogels for Multifunctional Sensors

**DOI:** 10.1002/advs.202416318

**Published:** 2025-02-20

**Authors:** Yingxiang Huang, Yanzhao Yang, Cong Peng, Yu Li, Wei Feng

**Affiliations:** ^1^ Institute of Advanced Technology and Equipment Beijing University of Chemical Technology Beijing 100029 China; ^2^ School of Materials Science and Engineering Tianjin University Tianjin 300350 China

**Keywords:** eutectic solvents, eutectogels, hierarchical microstructure, modified AuNPs, multifunctional sensing

## Abstract

Eutectogels with inherent ionic conductivity, mechanical flexibility, environment resistance, and cost‐effectiveness have garnered considerable attention for the development of wearable devices. However, existing eutectogels rarely achieve a balance between strength, strain, and resilience, which are critical indicators of reliability in flexible electronics. Herein, poly(sodium styrenesulfonate) (PSS)‐modified gold nanoparticles (AuNPs) in eutectic solvents are synthesized, and PSS‐AuNP reinforced polyacrylic acid/polyvinylpyrrolidone (SAu‐PAA/PVP) eutectogel is successfully prepared. Through the coordination between AuNPs and the PAA/PVP polymer chains, the SAu‐PAA/PVP eutectogel exhibits significantly enhanced tensile strain (946%), mechanical strength (3.50 MPa), and resilience (85.3%). The high‐performance eutectogel was demonstrated as a flexible sensor sensitive to strain and temperature, and the AuNPs provided near‐infrared sensing capabilities. Furthermore, SAu‐PAA/PVP eutectogel inherits the benefits of ES, including anti‐drying and anti‐freezing properties (−77 °C). Moreover, the eutectogel is microstructured using a simple molding method, and the resulting hierarchical pyramid microstructured eutectogel functions as ionic dielectric layer in a pressure sensor. This sensor exhibits high sensitivity (37.11 kPa^−1^), low detection limit (1 Pa), a fast response rate (36/54 ms), and excellent reproducibility over 5000 cycles, making them reliable and durable for detecting small vibrations, with potential applications in precision machinery, aerospace, and buildings.

## Graphical abstract

AuNP‐reinforced conductive eutectogels with excellent strength, strain, and resilience are prepared through a novel AuNP synthesis and modification strategy. The high‐performance eutectogel can be used for temperature, NIR, and strain sensors. Moreover, a hierarchical microstructured pressure sensor based on this eutectogel exhibits high sensitivity at low pressure, which shows great potential for sensing subtle vibrations.

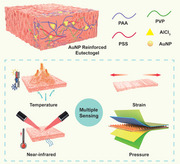

## 1 Introduction

With rapid advances in modern technology, flexible wearable electronics have shown tremendous potential in applications such as electronic skin,^[^ ^1^, ^2^, ^3^ ^]^ soft robotics,^[^ ^4^, ^5^, ^6^ ^]^ and health monitoring systems.^[^ ^7^, ^8^, ^9^ ^]^ As a core component of flexible electronics, sensors play a crucial role in shaping the functional design and future development of wearable devices. Flexible sensors, a groundbreaking technology, have garnered significant research interest and commercial adoption due to their ability to bend, stretch, and twist.^[^ ^10^, ^11^ ^]^ Compared to traditional rigid sensors, flexible sensors offer notable advantages, including enhanced flexibility, reduced weight, thinner profiles, and improved biocompatibility.^[^ ^12^, ^13^, ^14^ ^]^ Recently, ionic conducting eutectogels have attracted enormous attention as an alternative to conventional hydrogels and costly ionogels for flexible sensing applications.^[^ ^15^, ^16^, ^17^ ^]^ Its unique gel structure is derived from the phenomenon of eutectic solvents, where multiple components can form a mixture with optimal stability under specific temperatures and compositional conditions.^[^ ^18^, ^19^ ^]^ When embedded in a 3D polymer network, eutectic solvents form eutectogels that provide abundant ion‐conducting channels and impart exceptional mechanical tunability to the material. By varying the composition of eutectic solvents and polymer networks, the physicochemical properties of eutectogels can be customized to meet the requirements of diverse application scenarios.^[^ ^20^ ^]^ Consequently, the eutectogels are cost‐effective, easy to fabricate, and capable of maintaining stability under extreme environmental conditions, such as high temperatures, low humidity, and even outer space environments.^[^ ^21^, ^22^, ^23^, ^24^ ^]^ With the in‐depth research, the preparation methods and properties of eutectogels have been continuously improved, and their application potential has been continuously expanded, making them one of the research hotspots in the field of wearable devices.

The mechanical properties of eutectogels have been extensively studied, as they play a critical role in determining sensing performance.^[^ ^25^, ^26^, ^27^ ^]^ However, balancing strength, strain, and resilience in eutectogel materials remains a longstanding challenge, with most eutectogels meeting only one of these criteria. High resilience requires strong cross‐linking within the gel matrix, enabling the material to recover its original state through entropy‐driven processes after a force is released. Various strategies, such as covalent chemical cross‐linking,^[^ ^28^ ^]^ electrostatic interactions,^[^ ^29^ ^]^ metal coordination,^[^ ^30^ ^]^ and abundant hydrogen bonds,^[^ ^31^, ^32^ ^]^ impart resilience to eutectogels. Despite these efforts, their mechanical strength typically remains low, often below 0.5 MPa,^[^ ^33^, ^34^, ^35^ ^]^ limiting their structural versatility and suitability for intricate designs. Notably, a polyvinyl acetate eutectogel achieved a strength of 31.4 MPa due to the dense distribution of crystalline domains and hydrogen bond cross‐linking networks formed during freeze‐thaw cycling.^[^ ^36^ ^]^ And Wang et al.^[^ ^37^ ^]^ developed an ultra‐robust, subzero‐healable glassy polymer by incorporating polyphenol nano‐assemblies with numerous end groups into eutectogels. However, these high‐strength eutectogels generally exhibit significant hysteresis loops in their stress–strain curves, indicative of substantial residual strain. This residual strain compromises the reproducibility and baseline stability of sensors derived from such materials, leading to poor signal reliability.^[^ ^38^ ^]^ Therefore, developing eutectogels that simultaneously exhibit high strength, strain, and resilience remains a critical and urgent challenge to enhance the reliability and performance of eutectogel‐based sensors.

**Figure 1:**
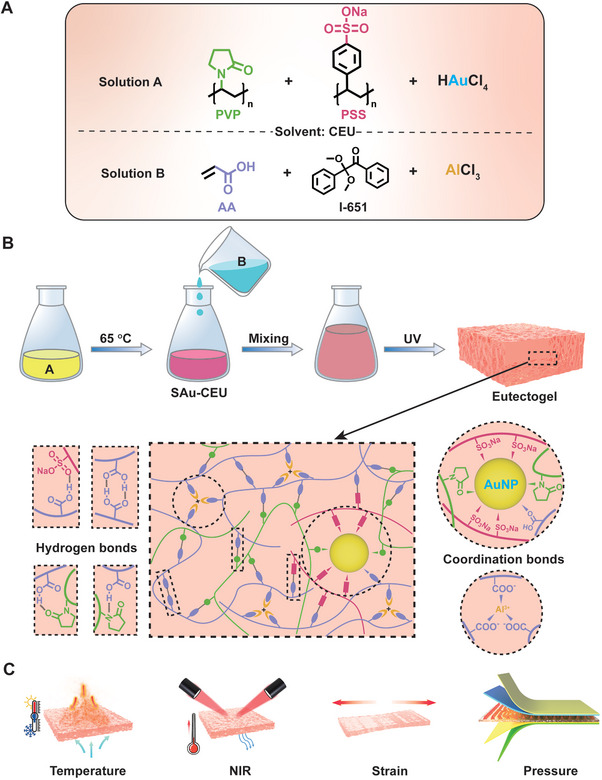
Preparation of SAu‐PAA/PVP eutectogels. A) Composition of solutions A and B. B) Preparation process and structural characterization of the SAu‐PAA/PVP eutectogels. C) Illustrative applications of SAu‐PAA/PVP eutectogels as flexible sensors for temperature, near‐infrared (NIR), strain, and pressure sensing.

Gold nanoparticles (AuNPs) are prone to aggregation or precipitation due to their elevated surface energy, Van der Waals interactions, and environmental factors such as ionic strength and pH variations, resulting in pronounced instability. Consequently, their incorporation into gels poses significant challenges and is rarely reported in the literature.^[^ ^39^, ^40^, ^41^, ^42^ ^]^ In this study, we synthesized a poly(sodium styrenesulfonate) (PSS)‐modified AuNP solution in a choline chloride (ChCl), ethylene glycol (EG), and urea ternary eutectic solvent (CEU), referred to as SAu‐CEU. The SAu‐CEU was stabilized by PSS, which, when mixed with a eutectogel prepolymer, enabled the successful synthesis of PSS‐AuNP reinforced polyacrylic acid/polyvinylpyrrolidone (SAu‐PAA/PVP) conductive eutectogels (**Figure** **1**). Multiple interactions in SAu‐PAA/PVP eutectogels (coordination between PAA and AlCl_3_, PAA and PVP with AuNPs, and hydrogen bonding interactions between the chains) result in high mechanical strength, excellent strain, outstanding resilience, and superior fatigue resistance. Due to the conductivity of AuNPs and CEU, the resulting eutectogels exhibit good electrical conductivity, while the low freezing point and volatility of the CEU impart excellent anti‐freezing and anti‐drying properties. The high‐performance eutectogel was demonstrated as a flexible sensor capable of detecting strain and temperature, with the incorporated AuNPs providing near‐infrared (NIR) sensing capability. Furthermore, the SAu‐PAA/PVP eutectogel was used to fabricate an innovative hierarchical pyramid‐structured pressure sensor, which exhibited higher sensitivity and a broader detection range compared to sensors with homogeneous pyramid structures. For a more visual demonstration, the ability of the pressure sensor to detect small vibrations was showcased by measuring the vibration waveforms of different drums in a resonant soundbox.

## 2 Results and Discussion

### 2.1 Preparation and Characterization of SAu‐PAA/PVP Eutectogels

The conductive eutectogels were prepared using a three‐step process. First, the CEU solvent, consisting of ChCl, EG, and urea, was used to prepare two solutions: solution A (containing PSS/PVP/HAuCl_4_·3H_2_O) and solution B (containing acrylic acid (AA), 2,2‐dimethoxy‐2‐phenylacetophenone (Irgacure 651), and AlCl_3_). The components were stirred thoroughly to ensure complete dissolution (Figure 1A). Next, solution A was placed in an oil bath and heated at 65 °C for 10 h, resulting in the formation of purplish‐red SAu‐CEU. Finally, the PAA prepolymer (solution B) was added to the SAu‐CEU to form a homogeneous solution, which was exposed to a 365 nm UV lamp to produce the SAu‐PAA/PVP eutectogels (Figure 1B).

**Figure 2:**
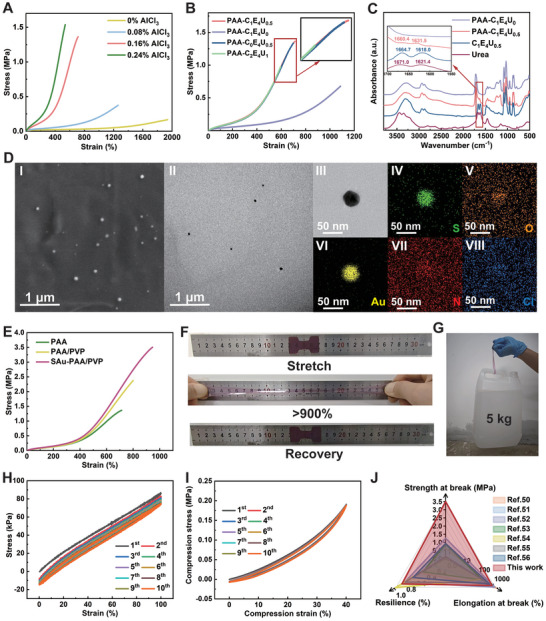
Optimization and characterization of SAu‐PAA/PVP eutectogels. A) Tensile stress–strain curves of PAA eutectogels with varying AlCl_3_ concentrations. B) Tensile stress–strain curves of PAA eutectogels with different CEU ratios. C) FTIR spectra of PAA‐C_1_E_4_U_0_, PAA‐C_1_E_4_U_0.5_, C_1_E_4_U_0.5_, and urea. D) TEM and SEM images of SAu‐PAA/PVP eutectogel and EDS maps of an individual AuNP. E) Tensile stress–strain curves of PAA, PAA/PVP, and SAu‐PAA/PVP eutectogels. Demonstration of F) tensile elongation and G) tensile strength of the SAu‐PAA/PVP eutectogel. H) Loading‐unloading (100% tensile strain) and I) compression‐relaxation (a compressive strain of 40%) of an SAu‐PAA/PVP eutectogel. J) A comparison of the mechanical properties of the SAu‐PAA/PVP eutectogel with other eutectogels was reported in the literature.

First, a PAA eutectogel was used to optimize the amount of AlCl_3_ and the CEU ratio in the PAA prepolymer. Similar to PAA hydrogels, the oxygen atoms in the carboxyl groups of the PAA chain have unpaired electrons that can coordinate with the empty orbitals of dissociated Al^3^⁺ ions, forming coordination bonds that aid in cross‐linking.^[^ ^43^, ^44^, ^45^ ^]^ As shown in the stress–strain curve of the PAA eutectogel (**Figure** **2**A), the tensile strength increased with the addition of AlCl_3_, demonstrating its cross‐linking effect. Based on these results, 0.16% AlCl_3_ (molar percentage relative to AA) was selected as it provided the best balance between strength and stretchability. Four CEU ratios were tested to determine the appropriate CEU ratio for balancing the mechanical properties and electrical conductivity of the eutectogel (Figure S1, Supporting Information). The results in Figure 2B indicate that the tensile strength of PAA eutectogels containing urea was significantly higher than that of PAA‐C_1_E_4_U_0_. A possible explaination is that  urea can combine with H⁺ ions released from PAA, raising pH of the solution. The increased pH causes the PAA chains to generate more ─COO^−^ groups, which form coordination bonds with Al^3+^, thereby enhancing the cross‐linking effect of AlCl_3_ with the PAA chain. Supporting this, we observed that the pH of PAA prepolymer solutions containing urea was higher than that of PAA‐C_1_E_4_U_0_ (Figure S2, Supporting Information), confirming the role of urea role in regulating the solution pH. To further validate this concept, the Fourier transform infrared (FTIR) spectra of the eutectogel were analyzed (Figure 2C). In C_1_E_4_U_0.5_, urea forms hydrogen bonds with ChCl, shifting the C═O stretching vibration to lower wavenumber (1671.0–1664.7 cm^−1^) along with the N─H bending vibration (1621.4–1618.0 cm^−1^). However, in PAA‐C_1_E_4_U_0.5_, the protonation of the urea ─NH_2_ group by H^+^ ions increased the strength and rigidity of the N─H bonds, shifting the N─H bending vibration to a higher wavenumber (1631.5 cm^−1^). Protonation also redistributed the electron density, weakening the strength of the C═O bond and causing the C═O stretching vibration to shift to a lower wavenumber (1660.4 cm^−1^). Compared to PAA‐C_0_E_4_U_0.5_, the addition of ChCl significantly improved the ionic conductivity of PAA‐C_1_E_4_U_0.5_. The characteristic peak of Cl^−^ in ChCl is observed at 952.7 cm^−1^ in the FTIR spectrum of PAA‐C_1_E_4_U_0.5_ (Figure S3A, Supporting Information), proving the presence of ChCl. However, excessive urea addition increased the viscosity of the system, which degrades the tensile strength and electrical conductivity, and characteristic FTIR peak intensities of ChCl and urea in PAA‐C_2_E_4_U_1_ were enhanced by doubling the amounts of ChCl and urea (Figure S3B, Supporting Information). Based on these observations, a ChCl: EG: urea ratio of 1: 4: 0.5 was selected for preparing the optimal CEU in this study.

In the synthesis of SAu‐CEU, the urea in the CEU slowly decomposes into ammonia, which acts as a reducing agent to reduce HAuCl_4_·3H_2_O at 65 °C without requiring additional additives.^[^ ^46^ ^]^ During the growth of AuNPs, ─SO^3−^ in PSS forms S─Au coordination bonds with AuNPs, while the C─N and C═O groups in PVP form weak coordination bonds on the surface of AuNPs, facilitating adsorption.^[^ ^47^, ^48^, ^49^ ^]^ Furthermore, due to the abundant hydrogen bonding interactions between the EG, urea, and ChCl in the CEU, a highly structured supramolecular solvent is created, enabling the self‐assembly and stabilization of AuNPs. To demonstrate the reducing property of urea and the stabilizing effect of the CEU, a series of solutions were designed (Table S1, Supporting Information). As shown by the experimental results, no AuNPs formed in solutions lacking urea (Figure S4, Supporting Information). The UV–visible–NIR  absorption spectra (Figure S5, Supporting Information) show an absorption peak of AuNPs only in solutions containing urea. Compared with EG/urea, the AuNP absorption peak was stronger in EG/urea/ChCl because ChCl formed abundant hydrogen bonds with EG and urea, generating and stabilizing more AuNPs. In H_2_O solutions, the addition of ChCl negatively affected AuNP stability, indicating that the hydrogen‐bonding supramolecular effect could not be established. The SAu‐CEU solution darkened with increasing concentrations of HAuCl_4_·3H_2_O (Figure S6, Supporting Information), and transmission electron microscope (TEM) images (Figure S7, Supporting Information) revealed that AuNPs in SAu‐CEU were well‐dispersed when the concentration of HAuCl₄·3H₂O was between 1 and 10 mg. Statistical analysis showed that the size of the AuNPs increased with higher HAuCl₄·3H₂O concentrations, consistent with the UV–visible–NIR absorption spectra (Figure S8, Supporting Information). However, When the concentration of HAuCl₄·3H₂O exceeded 15 mg, the system destabilized, and significant AuNP aggregation was observed. The UV–visible–NIR absorption peaks for SAu_15_‐CEU broadened and shifted toward higher wavelengths due to AuNP aggregation. Energy dispersive spectroscope (EDS) elemental maps (Figure S9, Supporting Information) confirmed the presence of Au, S, N, O, and Cl on the surfaces of the AuNPs. These results demonstrate that large amounts of PSS and PVP on the AuNP surfaces prevent agglomeration. Additionally, urea and ChCl from the CEU eutectic solvent adsorbed onto the AuNP surfaces, further stabilizing them through the formation of a hydrogen‐bonding network.

The PAA prepolymer contains abundant anions and cations, while the unmodified AuNP solution exhibits weak resistance to interference. Adding the PAA prepolymer to the AuNP solution disrupts the equilibrium state, causing the AuNPs to rapidly agglomerate (Figure S10, Supporting Information). In contrast, the SAu‐CEU solution significantly delays the effects of ions after mixing, providing sufficient time for subsequent reactions and enabling the successful synthesis of AuNP composite eutectogels (Figure S11, Supporting Information). SAu_x_‐PAA/PVP eutectogel composites with varying AuNP contents were prepared using different concentrations of SAu‐CEU, with the observed color changes consistent with those of the corresponding SAu_x_‐CEU solutions (Figure S12A, Supporting Information). The UV–visible–NIR absorption spectra (Figure S12B, Supporting Information) show that the absorption peaks of the SAu_x_‐PAA/PVP eutectogels align with those of the corresponding SAu_x_‐CEU solutions, indicating that the AuNPs remain stable within the eutectogel matrix. The SEM and TEM images of SAu_10_‐PAA/PVP (Figure 2DI‐II) reveal uniformly dispersed AuNPs, suggesting strong interfacial interactions within the eutectogel and the formation of a homogeneous composite material. The corresponding EDS maps of an individual AuNP in SAu_10_‐PAA/PVP (Figure 2DIII‐VIII) show that sulfur aggregation is clearly visible on the AuNP surface, confirming the formation of strong Au─S coordination bonds between PSS and the AuNPs, which play a protective role. Furthermore, the accumulation of nitrogen and oxygen on the AuNPs indicates that PVP and PAA chains also coordinate with the AuNPs.

To evaluate the role of each component in the SAu‐PAA/PVP eutectogel, PAA and PAA/PVP eutectogels were prepared for comparison. In the PAA/PVP eutectogel, PVP acts as a secondary network structure, enhancing the mechanical properties of the PAA eutectogel (Figure 2E). For SAu‐PAA/PVP eutectogels, the addition of AuNPs enables the C═O and C─N groups in PVP, as well as the ─COOH groups in PAA, to form coordinated cross‐linking points on the AuNP surfaces. Therefore, the AuNPs in SAu‐PVP/PAA eutectogel strengthens the PAA/PVP polymer network structure. Increasing the AuNP content provides more cross‐linking points, resulting in improved mechanical properties of the eutectogels (Figure S13, Supporting Information). Among the samples, SAu_10_‐PAA/PVP demonstrated the highest tensile elongation (946.1%) and tensile strength (3.504 MPa). However, further increasing the AuNP concentration led to the formation of AuNP agglomerates, which reduced the mechanical properties of the eutectogels, as observed in SAu_15_‐PAA/PVP. Consequently, SAu_10_‐PAA/PVP was selected as the optimal system. Figure 2F,G highlights the superior tensile strain and strength of the SAu_10_‐PAA/PVP eutectogel.

The cycling stability and resilience of eutectogels are critical factors determining the durability of flexible sensors. To assess these properties, 10 consecutive loading–unloading cycles (at 100% tensile strain) and compression–relaxation cycles (at 40% compressive strain) were performed on SAu‐PAA/PVP eutectogel samples (Figure 2H,I). During deformation, the hydrogen and coordination bonds dissipate energy and rapidly reform upon unloading, preventing structural damage from external forces. Compared with PAA/PVP eutectogels (Figure S14, Supporting Information), the SAu‐PAA/PVP eutectogels exhibited smaller hysteresis loops, indicating that the coordination bonds formed by the AuNPs significantly enhance cycling performance and resilience. The resilience of the SAu‐PAA/PVP eutectogel was calculated (Figure S15, Supporting Information) to be 85.3% and 84.2% (75.6% and 67.9% for PAA/PVP) in the first loading‐unloading cycle at a strain of 100% and compression of 40%, respectively. In comparison, the mechanical properties of eutectogels reported in the literature are summarized in Figure 2J (Table S2, Supporting Information).^[^ ^50^, ^51^, ^52^, ^53^, ^54^, ^55^, ^56^ ^]^ These results highlight the superiority of the SAu‐PAA/PVP eutectogel in terms of tensile strength, elongation, and resilience.

### 2.2 Environment Resistances of SAu‐PAA/PVP Eutectogels

**Figure 3:**
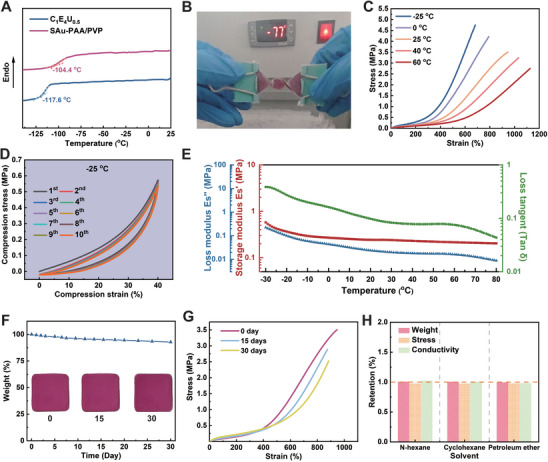
Anti‐freezing, anti‐drying, and solvent resistance performance of SAu‐PAA/PVP eutectogel. A) DSC curve of CEU eutectic solvent and SAu‐PAA/PVP eutectogel. B) Photograph of twisting SAu‐PAA/PVP eutectogel after cooling to −77 °C. C) Tensile stress–strain curves of SAu‐PAA/PVP eutectogel at various temperatures. D) Stress–strain curves of SAu‐PAA/PVP eutectogel at −25 °C during 10 consecutive compression cycles. E) Compressive storage modulus (Es′), loss modulus (Es″), and loss tangent (tan δ) of the SAu‐PAA/PVP eutectogel across a temperature range from −30 to 80 °C. F) Weight loss curve (inset: photographs) and G) tensile stress–strain curves of the SAu‐PAA/PVP eutectogel stored at ambient conditions (25 °C, 30% humidity). H) Weight, stress, and conductivity retention of the SAu‐PAA/PVP eutectogel after immersion in nonpolar solvents (N‐hexane, cyclohexane, and petroleum ether) for 24 h.

The mechanical properties of conventional hydrogels significantly deteriorate at subzero temperatures due to the freezing of water, which disrupts the internal structure and reduces the flexibility of the polymer chains of hydrogels. In contrast, the molecules in CEU inhibit the crystallization and solidification of solvent molecules at low temperatures through strong hydrogen bonding interactions between ChCl, EG, and urea, providing exceptional anti‐freezing properties. Differential scanning calorimetry (DSC) results (**Figure** **3**A) confirm that the glass transition temperature (Tg) of the SAu‐PAA/PVP eutectogel is −104.4 °C, slightly higher than that of the corresponding CEU eutectic solvent (−117.6 °C). As shown in Figure 3B; Movie S1 (Supporting Information), the SAu‐PAA/PVP eutectogel exhibits excellent freezing resistance, remaining flexible and capable of being twisted even after being placed in a refrigerator at −77 °C for 30 min. Figure 3C illustrates the tensile properties of the SAu‐PAA/PVP eutectogel at various temperatures. At 25 °C, the eutectogel achieved an impressive strain of 946.1%. Even at −25 °C, the strain remained as high as 682.0%. Notably, the tensile strength increased from 3.504 to 4.743 MPa upon cooling to −25 °C. Generally, as temperature increases, elongation at break increases while tensile strength decreases. This behavior is attributed to the reduced solvent viscosity and enhanced mobility of polymer chains at elevated temperatures. Similarly, the SAu‐PAA/PVP eutectogel demonstrates good resilience during compression‐relaxation cycles at −25 °C (Figure 3D). Dynamic mechanical analysis (DMA) under compression (Figure 3E) further confirms that the SAu‐PAA/PVP eutectogel maintains good elasticity across a temperature range from 70 to −30 °C, with no abrupt changes in the storage and loss modulus. The decrease in mechanical properties at high temperatures is attributed to a reduction in the loss modulus. At temperatures exceeding 70 °C, thermogravimetric analysis (TGA) (Figure S16, Supporting Information) indicates a gradual evaporation of EG, resulting in a significant reduction in the eutectogel modulus.

Excellent water retention is crucial for the development of stable sensor systems. Conventional hydrogels are susceptible to dehydration in dry environments, leading to a drastic decline in performance. Similar to ionic liquids, CEU eutectic solvents have low saturation vapor pressures and are expected to exhibit strong resistance to drying. To evaluate this property, the SAu‐PAA/PVP eutectogel was stored in an airtight container under controlled temperature and humidity conditions (25 °C, 30%), and changes in the sample were monitored. As shown in Figure 3F, the mass loss of the SAu‐PAA/PVP eutectogel was only 7.22% after 30 days of storage, with no noticeable change in the shape or color of the sample. Tensile tests conducted during storage (Figure 3G) revealed that the elongation at break and tensile strength of the eutectogel decreased by only 6.61% and 27.92%, respectively, after 30 days. The reduction in mechanical properties is likely due to the volatilization of EG, which alters the CEU eutectic solvent ratio and affects the coordination cross‐linking between AlCl_3_ and PAA. These results demonstrate that the SAu‐PAA/PVP eutectogel has excellent resistance to drying, making it a strong candidate for flexible sensing applications in harsh environments.

In addition to its resistance to drying, the SAu‐PAA/PVP eutectogel exhibits good stability in nonpolar solvents due to its exclusively polar hydrophilic components. When immersed in three nonpolar solvents (n‐hexane, cyclohexane, and petroleum ether) for 24 h, the eutectogel showed no significant changes in appearance (Figure S17A, Supporting Information). Furthermore, the weight, conductivity, and tensile properties (Figure 3H; Figure S17B, Supporting Information) remained nearly identical to the original values. The exceptional stability of the SAu‐PAA/PVP eutectogel in nonpolar solvents enhances its potential for use in flexible sensing systems, particularly in challenging operational environments.

### 2.3 Temperature, NIR, and Strain Sensing Performances

Eutectic solvents are inherently ionically conductive, allowing eutectogels based on this solvent system to exhibit conductivity without the addition of salts. The conductivity of the SAu‐PAA/PVP eutectogel was enhanced to 0.792 mS cm^−1^ compared to PAA and PAA/PVP eutectogels (Figure S18, Supporting Information), owing to the excellent electron‐transport properties and electrical conductivity of AuNPs. This makes the eutectogel suitable for use as a resistivity‐type sensor.

**Figure 4:**
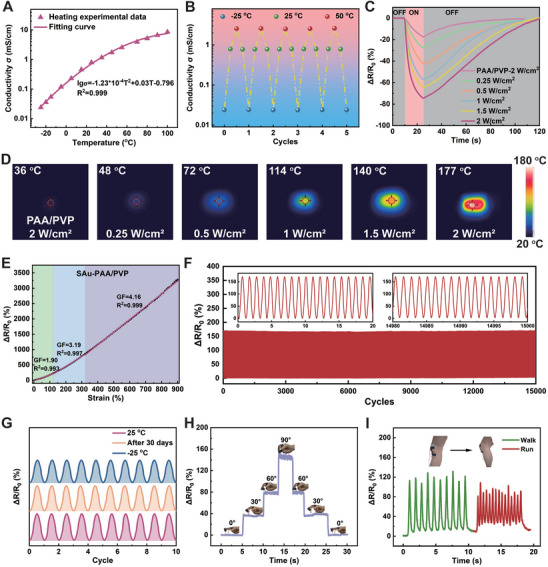
Temperature, NIR, and strain sensing performance. A) Temperature‐dependent conductivity of the SAu‐PAA/PVP eutectogel. B) ΔR/R_0_ response of the SAu‐PAA/PVP eutectogel during cyclic tests at −25, 25, and 50 °C. C) Photoelectric responsivity and D) infrared thermal images of the SAu‐PAA/PVP eutectogel under illumination with various powers of 808 nm NIR light. E) ΔR/R_0_ of the SAu‐PAA/PVP eutectogel as a function of strain. F) Durability of the sensor over 15000 stretching cycles at 50% strain. G) ΔR/R_0_ of the SAu‐PAA/PVP eutectogel under 50% strain cyclic characterization at 25, −25 °C, and after 30 days of storage at ambient conditions (25 °C, 30%). ΔR/R_0_ curves of the sensor in response to movements of different body parts: H) finger and I) knee.

Temperature, NIR, and strain sensors were assembled by connecting two electrodes to the ends of a rectangular SAu‐PAA/PVP eutectogel sample. The remarkable properties of the eutectogel make it ideal for developing temperature sensors that are highly stable across a wide operating temperature range. Elevated temperatures enhance ion mobility within the eutectogel, while the dissociation of ChCl at higher temperatures increases the carrier concentration. These effects lead to a significant positive correlation between eutectogel conductivity and temperature, making it suitable for temperature sensing (**Figure** **4**A). Although the conductivity of AuNPs is negatively correlated with temperature, the temperature‐dependent variation in ionic conductivity is significantly stronger than that of AuNPs. Consequently, the conductivity of the SAu‐PAA/PVP eutectogel is primarily determined by ionic conductivity. Cyclic conductivity tests were conducted at three temperature points (−25, 25, and 50 °C) to evaluate temperature sensing performance (Figure 4B). The conductivities at each temperature remained stable throughout the cycling process, demonstrating good reproducibility. To further illustrate its temperature sensing capabilities, the SAu‐PAA/PVP eutectogel was transferred from a 25 °C environment to 70 °C and then immediately placed in a 10 °C condition after stabilization. The resistance change curve (Figure S19, Supporting Information) confirms its stable temperature sensing performance.

For spherical solid AuNPs, only those larger than 50 nm or aggregates exhibit strong NIR absorption.^[^ ^57^ ^]^ Approximately 15% of the AuNPs in SAu_10_‐CEU (Figure S6D, Supporting Information) and SAu_10_‐PAA/PVP eutectogel (Figure 2DI‐II) were larger than 50 nm, with a small number of aggregates containing 2–3 AuNPs. These AuNPs generate a strong surface plasmon resonance (SPR) phenomenon under NIR irradiation, converting incident NIR light into thermal energy. The UV–visible–NIR transmission spectrum (Figure S12B, Supporting Information) also confirms that the SAu‐PAA/PVP eutectogel exhibits absorption activity in the NIR region. Therefore, the temperature of the SAu‐PAA/PVP eutectogel increases significantly under NIR irradiation. As discussed earlier, the conductivity of the eutectogel is positively correlated with temperature, meaning that NIR stimulation results in a measurable change in resistance. To explore the potential of the SAu‐PAA/PVP eutectogel as a NIR‐light‐responsive photothermoelectric sensor, its photothermoelectric responsiveness was investigated by recording resistance and temperature changes under different NIR power levels using a digital bridge and infrared thermography. Figure 4C shows that the 808 nm NIR response of the SAu‐PAA/PVP eutectogel with AuNPs was significantly enhanced compared to that of the PAA/PVP eutectogel. The ΔR/R₀ response steadily increased with higher NIR power, attributed to the uniform distribution of AuNPs within the eutectogel, which enabled stable photothermal conversion. Infrared thermography was used to capture temperature changes in the eutectogel under NIR irradiation (Figure 4D). The maximum temperature of the SAu‐PAA/PVP eutectogel reached 177 °C after 15 s of NIR irradiation at 2.0 W cm^−2^, while the PAA/PVP eutectogel only reached 36 °C, demonstrating the highly efficient photothermal conversion ability of AuNPs. To visualize the NIR response of the eutectogel, wires attached to the SAu‐PAA/PVP eutectogel were connected to an LED light (Figure S20, Supporting Information), which glowed brighter after NIR irradiation. This indicates that the resistance of the SAu‐PAA/PVP eutectogel decreases under NIR irradiation. These results suggest that the SAu‐PAA/PVP eutectogel sensor has potential applications in temperature and NIR detection systems.

An applied tensile strain increases the length of the eutectogel while simultaneously decreasing its cross‐sectional area, resulting in an increase in resistance. The gauge factor (GF), defined as the change in relative resistance with the applied strain, is a critical parameter for evaluating the sensitivity of strain sensors. Based on its high GF values, the sensor demonstrated excellent sensitivity (Figure 4E), with a GF of 1.90 at 0–120% strain, 3.19 at 120–320% strain, and 4.16 at 320–900% strain. Due to the electronic conductivity of AuNPs, the sensitivity of the SAu‐PAA/PVP eutectogel strain sensor was higher than that of PAA and PAA/PVP (Figure S21, Supporting Information). By applying a transient tensile strain to the sensor (Figure S22, Supporting Information), fast response and recovery times of 0.125 and 0.15 s, respectively, were achieved. When the SAu‐PAA/PVP eutectogel sensor was repeatedly stretched to 100% strain for 15 000 cycles, it produced nearly identical resistance signals (Figure 4F), indicating excellent stability and reversibility. More importantly, the outstanding anti‐drying and anti‐freezing properties of the SAu‐PAA/PVP eutectogel enabled the strain sensor to maintain stable resistance signals at −25 °C or after 30 days of storage under ambient conditions (Figure 4G).

A wearable strain sensor based on the SAu‐PAA/PVP eutectogel was fabricated to evaluate its potential for monitoring human motion. The eutectogel sensor was attached to a finger to monitor movement (Figure 4H). As the bending angle of the finger increased, the strain applied to the eutectogel caused a higher ΔR/R_0_ signal. Repeated bending of the finger at the same angle produced highly consistent resistance signals, demonstrating the high reproducibility of the sensor. Similarly, when the sensor was attached to the wrist, elbow, and neck (Figure S23, Supporting Information), their movements were effectively monitored with high repeatability. Furthermore, when the sensor was attached to the knee joint (Figure 4I), it successfully monitored step frequency and distinguished between walking and running.

### 2.4 Fabrication and Performance of Hierarchical Pyramid Microstructure Pressure Sensor

One effective method for improving the sensitivity of pressure sensors is introducing various micropatterns into the electrodes or dielectric layers.^[^ ^58^, ^59^, ^60^, ^61^ ^]^ The geometry of micropatterned materials is highly deformable under external forces, resulting in significant changes at the points of contact or in the areas between sensing elements. This deformation leads to enhanced sensitivity or larger sensing ranges. In this study, a hierarchical pyramidal microstructured surface was innovatively designed, and the fabrication process is illustrated in Figure S24 (Supporting Information).

**Figure 5:**
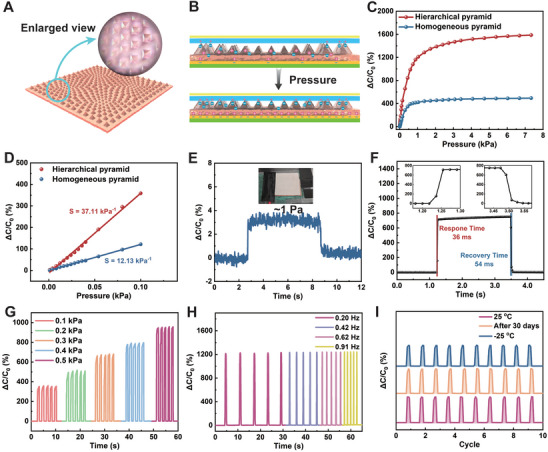
Fabrication and performance of hierarchical pyramid microstructured pressure sensor. A) SAu‐PAA/PVP eutectogel with hierarchical pyramid microstructures and magnified images of the microstructures. B) Schematic Illustration of the ionic pressure sensing mechanism for the hierarchical pyramid microstructured device. C) ΔC/C_0_ and D) enlarged view (0 to 0.1 kPa) of sensors with hierarchical and homogeneous pyramid microstructured dielectric layers. E) ΔC/C_0_ with an applied pressure of 1 Pa, F) response and recovery times, G) ΔC/C_0_ under cyclic pressure application, and H) ΔC/C_0_ to compressive force at different frequencies for the hierarchical pyramid microstructured sensor. I) ΔC/C_0_ of the hierarchical pyramid microstructured sensor under 0.5 kPa cyclic characterization at 25, −25 °C, and after 30 days of storage under ambient conditions (25 °C, 30%).

To fabricate the microstructured eutectogel, the prepared eutectogel prepolymer solution was poured into a Cu mold surrounded by a silicone pad and cured using a 365 nm UV lamp. The cured sheet was then peeled off the mold and cut to obtain the desired microstructured eutectogel. Unlike the homogeneous pyramid microstructure (Figure S25A, Supporting Information), the hierarchical design features a stepped microstructure (**Figure** **5**A; Figure S25B, Supporting Information), with a circular perimeter and two vertical columns in the center acting as supports. The triangular pyramids in each quarter progressively decrease in size from the perimeter to the center. Photographs and magnified views of the hierarchical and homogeneous pyramid microstructured eutectogels (Figure S26, Supporting Information) reveal uniformly arranged arrays with well‐defined edges and a clear gradient in pyramid sizes in the hierarchical structure. The microstructured eutectogels were subsequently used to assemble capacitive pressure sensors (Figure S27, Supporting Information). Further details on the fabrication of the microstructured ionic dielectric layers and capacitive pressure sensors are provided in the Experimental Section (Supporting Information).

To elucidate the underlying principle of capacitive pressure sensing, cross‐sections of 1/4 of the microstructured sensor are shown for visualization (Figure 5B). Upon ionic–electronic contact, interfacial electric double layers (EDLs) are immediately established, resulting in high capacitance. The capacitance of the EDL is directly proportional to the contact area between the electrodes and the eutectogel and inversely proportional to the distance between the electrodes. Owing to the unique hierarchical pyramid design, the eutectogel layer initially contacts the top electrode only at a few pyramidal vertices. Thus, the initial capacitance (C_0_) is very small, ≈30 pF at 100 kHz. Compared to the homogeneous structure, the layered hierarchical eutectogel deforms more significantly under compression, increasing the rate of change in the contact area. This results in a substantial increase in EDL capacitance (ΔC) and high sensor sensitivity, expressed as S = ΔC/C_0_P, where P is the applied pressure. At higher pressures, the small 4 × 4 pyramid arrays in the central regions of each quarter continue to deform, maintaining pressure detection capability. As shown in Figure 5C, the homogeneous pyramid structure exhibits premature pressure saturation, whereas the hierarchical pyramid structure retains considerable pressure detection capability up to 7 kPa. Linear fitting of the capacitance curve in the low‐pressure region (Figure 5D) indicates that the sensitivity of the hierarchical pyramid structure (37.11 kPa^−1^) is significantly higher than that of the homogeneous pyramid structure (12.13 kPa^−1^) at low pressures (0 to 0.1 kPa), demonstrating its potential for detecting small perturbations. To validate this, a square piece of paper (≈1 Pa) was placed on the pressure sensor, resulting in a clear signal change (Figure 5E). Further experiments were conducted to evaluate the response rate, repeatability, and reliability of the hierarchical pyramid‐structured pressure sensor. Analysis of the signals during the loading and unloading phases (Figure 5F) revealed response and relaxation times of 36 and 54 ms, respectively, which are comparable to the human knee‐jerk reflex rate (≈50 ms). Figure 5G shows that the on/off ratio of the capacitance increases significantly and exhibits a step‐like characteristic as the applied pressure increases, indicating that the device can quickly recognize different pressure levels. The capacitive signal was frequency‐independent at the same pressure, further highlighting the sensor's high reliability (Figure 5H). Additionally, the steady performance over 5000 consecutive pressure cycles demonstrates the sensor's excellent stability (Figure S28, Supporting Information). Furthermore, owing to the environmental resistance of the SAu‐PAA/PVP eutectogel, the signal and baseline values remained stable at −25 °C or after 30 days of storage under ambient conditions (Figure 5I). The fast response rate and excellent repeatability of the pressure sensor are attributed to the good compression elasticity and fatigue resistance of the SAu‐PAA/PVP eutectogel. As a result, the sensor can distinguish between pressing (stable capacitance change) and rapid knocking (capacitance spikes) (Figure S29, Supporting Information). Interestingly, the sensor also responded to and distinguished between different wind speeds in the range of 2–13 m s^−1^ (Figure S30A, Supporting Information). When the air was blown against the sensor, a clear change in the capacitance signal was observed (Figure S30B, Supporting Information). These capabilities demonstrate the sensor's considerable potential for monitoring minute disturbances.

### 2.5 Potential Applications of Pressure Sensors as Vibration Signal Monitor

**Figure 6:**
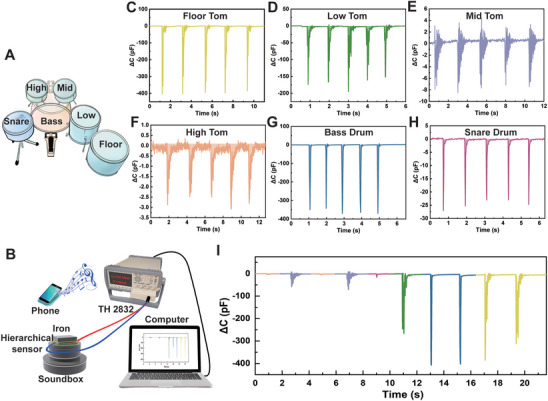
Applications of the pressure sensor for monitoring vibration signals. A) Illustration of the studied drum set. B) Schematic representation of the experimental setup for detecting vibrations using the pressure sensor. ΔC signals of the sensor during the playing of the C) floor tom, D) low tom, E) mid tom, F) high tom, G) bass drum, and H) snare drum. I) Vibration signals are recorded by the pressure sensor during the drum‐beating process.

The principle of a resonance soundbox is that audio is converted into mechanical vibrations, which are transferred to the surface of a medium (e.g., wooden desktop, glass). This enables the medium to resonate and produce melodious music. Unlike traditional sound systems, this type of soundbox does not use a diaphragm but relies on direct contact with hard objects to generate sound. While playing drums, the resonant sound produces varying vibration amplitudes and frequencies, depending on the unique vibration characteristics of each drum. Due to the high sensitivity and fast response speed of the hierarchical pyramid‐structured pressure sensor, it can capture these vibration changes and provide real‐time feedback through a capacitive signal. An illustration of the studied drum kit is shown in **Figure** **6**A, arranged from right to left as follows: tom‐tom drums (floor tom, low tom, mid tom, and high tom), bass drum, and snare drum. As depicted in Figure 6B, the sensor was placed on a resonance soundbox with a small square iron block positioned on top to transmit vibrations. The pressure sensor was connected to a TH2832 LCR digital bridge, and a computer displayed the sensor's capacitance changes in real time. A smartphone was paired with the resonance soundbox via Bluetooth to input commands for playing different drum sounds.

The pressure sensor successfully detected four distinct signals corresponding to the variable pitches produced by the tom‐tom drums in the resonant soundbox (Figure 6C–F). The floor tom, which produces the lowest pitch among the tom‐toms, generates strong vibrations, resulting in intense capacitive peaks in the sensor output. Similarly, the low tom, mid tom, and high tom drums, which produce progressively higher pitches, yielded capacitive peaks with decreasing amplitudes but increasing vibration frequencies. The bass drum, known for its deep and mellow bass effect, exhibited a peak amplitude similar to that of the floor tom but at a lower frequency (Figure 6G). In contrast, the snare drum, characterized by a crisp, loud, and high‐frequency sound, produced a relatively low peak amplitude (Figure 6H). To further validate the sensor's performance, we simulated the drum set and recorded the signals generated during the drum beating process (Figure 6I; Movie S2, Supporting Information). The vibrations captured during the beating process remained consistent with the initial recorded signals, demonstrating the reliability of the pressure sensor in detecting small vibrations.

## 3 Conclusion

In this study, we developed a novel strategy for synthesizing and modifying AuNPs to prepare SAu‐PAA/PVP conductive eutectogels. The stability of AuNPs modified by PSS was significantly enhanced in prepolymers, enabling the uniform distribution of AuNPs within the eutectogel. Through the coordination of AuNPs with PAA and PVP chains, SAu‐PAA/PVP eutectogels achieved a remarkable combination of excellent strength, high strain, and outstanding resilience, making them highly suitable for flexible strain sensors with exceptional stability and durability. Additionally, the temperature‐dependent ionic conductivity and the unique SPR phenomenon of AuNPs endowed the SAu‐PAA/PVP eutectogels with advanced temperature and NIR‐sensing capabilities. Importantly, the eutectogels inherited the advantages of CEU eutectic solvent, offering excellent anti‐drying and anti‐freezing properties, and remained highly reliable even after 30 days of storage under dry conditions or at −25 °C. Furthermore, we innovatively fabricated a pressure sensor using a hierarchical pyramid microstructured SAu‐PAA/PVP eutectogel as the ionic dielectric layer. This sensor demonstrated exceptional performance, including high sensitivity (37.11 kPa^−1^), a fast response rate (36/54 ms), a low detection limit (≈1 Pa), and excellent reproducibility over 5000 cycles. Its ability to detect subtle vibrations was validated by its connection to a resonant soundbox, highlighting its potential for monitoring critical component vibrations in industrial equipment, assessing aircraft vibration status, or evaluating the structural integrity of buildings and bridges. This study provides a novel approach for preparing AuNP‐reinforced eutectogels and highlights the unique benefits of the hierarchical pyramid microstructure. These findings offer valuable insights for designing future flexible electronics with enhanced functionalities and innovative applications.

## Conflict of Interest

The authors declare no conflict of interest.

## Supporting information



Supporting Information

Supplemental Movie 1

Supplemental Movie 2

## Data Availability

The data that support the findings of this study are available from the corresponding author upon reasonable request.
