# Supplementary material for: High Strength, Strain, and Resilience of Gold Nanoparticle Reinforced Eutectogels for Multifunctional Sensors
Source: Adv Sci (Weinh). 2025 Feb 20;12(15):2416318. doi: 10.1002/advs.202416318 (PMC12005770; doi:10.1002/advs.202416318)
Supplement: Supplementary file 1 — Supporting Information [file ADVS-12-2416318-s003.docx]

**Supporting Information**

**High Strength, Strain, and Resilience of Gold Nanoparticle Reinforced Eutectogels for Multifunctional Sensors**

*Yingxiang Huang, Yanzhao Yang, Cong Peng, Yu Li, and Wei Feng**

Y. Huang, C. Peng, Y. Li, W. Feng

Institute of Advanced Technology and Equipment, Beijing University of Chemical Technology, Beijing, China

E-mail: weifeng@tju.edu.cn (W.F.)

Y. Yang, W. Feng

School of Materials Science and Engineering, Tianjin University, Tianjin, China

E-mail: weifeng@tju.edu.cn (W.F.)

**Materials and methods**

**Materials:** Acrylic acid (AA), urea, AlCl_3_, poly(sodium styrenesulfonate) (PSS, Mw~1000,000), and polyvinylpyrrolidone (PVP, Mw~58,000) were purchased from Macklin. Choline chloride (ChCl), ethylene glycol (EG), and 2,2-dimethoxy-2-phenylacetophenone (Irgacure 651) were obtained from Aladdin. HAuCl_4_·3H_2_O was purchased from Tianjin Zancheng Co.

**Preparation of CEU eutectic solvent:** ChCl, EG, and urea were mixed in molar ratio of a:b:c, then stirred and heated to 80 °C until a transparent CEU solution was formed. This solution was denoted as C_a_E_b_U_c_, where a, b, c represent the molar ratios of ChCl, EG, and urea, respectively.

**Synthesis of PSS-modified AuNP solution (SAu_x_-CEU)****:** PVP (0.75 g), PSS (0.04 g), and HAuCl_4_·3H_2_O were added to 12.5 g of CEU and dissolved with stirring at 25 °C to form a homogeneous solution. The solution was reacted in an oil bath at 65 °C for 10 h to produce the PSS-modified AuNP solution, denoted as SAu_x_-CEU, where x represents the mass of HAuCl_4_·3H_2_O added (mg).

**Synthesis of SAu-PAA/PVP eutectogels:** The AA monomer was purified by spin distillation at 45 °C. AlCl_3_ was added to 12.5 g of CEU and stirred at 25 °C for 6 h until complete dissolution. Purified active monomers, AA (13 g) and Irgacure 651 (0.2 g), were added and stirred for 20 min to prepare the **PAA prepolymer.** The prepared SAu-CEU was quickly poured into the solution, and thorough stirring produced a precursor solution containing AuNPs. This precursor solution was poured into a specific silicone mold, placed in a vacuum chamber to remove air bubbles for 5 min, fixed between two glass plates, and illuminated with a 365 nm UV lamp for 15 min. After removing the glass plates and silicone molds, the SAu-PAA/PVP eutectogels were obtained. The size of the SAu-PAA/PVP eutectogel was adjusted by varying the shape and thickness of the silicone mold.

**Preparation of PAA eutectogels:** To optimize the AlCl_3_ and CEU eutectic solvent ratios in the PAA prepolymer, 12.5 g of pure CEU was added to the PAA prepolymer. PAA eutectogels were then prepared using the same synthesis method as described for SAu-PAA/PVP eutectogels.

**Preparation of PAA/PVP eutectogels:** For comparison, PVP (0.75 g) was dissolved in 12.5 g of CEU by stirring at 25 °C to form a homogeneous solution. This solution was then added to the PAA prepolymer. PAA/PVP eutectogels were subsequently prepared using the same synthesis method as described for SAu-PAA/PVP eutectogels.

**Fabrication of flexible strain/temperature/NIR sensors:** Rectangular eutectogel samples with dimensions of 4 cm × 1 cm were cut from a molded sheet with a thickness of 2 mm. Copper (Cu) wires were attached to Each end of the rectangular sample, and these wires were connected to a TH2832 LCR digital bridge to monitor the resistance (R) of the signal. The relative change in resistance ∆R/R_0_ = (R−R_0_)/R_0_, where R is the real-time resistance and R_0_ is the initial resistance.

**Fabrication of microstructured ionic dielectric layers:** A customized copper mold surrounded by a silicone ring was used; a uniformly dispersed SAu-PAA/PVP precursor solution was poured into the mold, vacuum-defoamed, fixed between two glass plates, and irradiated with a 365 nm UV lamp for 15 min. After removing the glass plates and mold, the microstructured SAu-PAA/PVP eutectogel ionic dielectric layer was obtained.

**Assembly of flexible capacitive pressure sensors:** A Cu membrane tape was cut into a 31 mm × 31 mm sample, and the adhesive side was applied to the flat surface of an SAu-PAA/PVP eutectogel dielectric layer. Then, a 2 mm × 2 mm 3M double-sided adhesive was applied to the center and the four corners of the ITO/PET conductive surfaces. The conductive ITO side was bonded to the microstructured surface of the SAu-PAA/PVP eutectogel. Finally, the Cu and ITO/PET electrodes of the capacitive flexible sensor were protected with insulating PET and PI films, respectively, to maintain good signal stability. The capacitance signal was monitored using a TH2832 LCR digital bridge (1 V, 100 kHz). The relative capacitance variation (∆C/C_0_) was calculated using ∆C/C_0_ = (C-C_0_)/C_0_, where C is the real-time capacitance and C_0_ is the initial capacitance.

**FTIR characterization:** Attenuated total reflection (ATR) FTIR spectra of the eutectogels were recorded using a Fourier transform spectrometer (Thermo Scientific Nicolet iS50) to analyze the functional groups and intermolecular interactions in the eutectogels. The spectra were collected over a scanning range of 3800–500 cm^-1^.

**UV-Visible-NIR Spectroscopy characterization:** The UV-visible-NIR absorption data of the AuNP solutions and eutectogels were obtained using a UV-visible-NIR spectrophotometer (SPC UV-2600i). CEU eutectic solvent containing AuNPs (diluted to 1/3) were measured using a 5 mm optical range quartz cuvette, while 2mm thick molded eutectogel sheets were analyzed directly. The measurements were performed over a wavelength range of 350-850 nm.

**Microscopy characterization:** The dispersion of AuNPs within the cross-section of the eutectogel sheets was analyzed using a Hitachi S4800 SEM. Because the AuNPs in the eutectogel provide high conductivity, SEM analysis was conducted without applying a gold coating. TEM images were acquired using a JEOL JEM-F200 instrument at an accelerating voltage of 200 kV. The elemental composition of the AuNP surface was determined using EDS. **AuNP solution:** A 0.1 mL aliquot of the AuNP solution was dropped onto a copper mesh and placed in an oven at 65°C to evaporate the liquid before testing. **Eutectogels:** the eutectogels were sliced into 50-100 nm thick sections using a cryo-ultramicrotome (Leica EM UC7) at -150 °C and transferred onto an ultrathin copper mesh for imaging.

**DSC characterization:** The freezing and glass transition temperatures of CEU and eutectogels were analyzed using a Netzsch DSC 3500 instrument. The temperature range was set from 50 to -140°C with a cooling rate of 5°C min^-1^ under a N_2_ atmosphere flow.

**TGA** **measurement:** The thermal stability of the eutectogel was evaluated using a HITACHI STA200 simultaneous thermal analyzer. The measurements were conducted at a heating rate of 5 °C min^-1^ from room temperature to 600 °C under a N_2_ atmosphere flow.

**Mechanical tests:** Room-temperature mechanical tests were performed using a universal electronic testing machine (SUNS, UTM2203X) equipped with a 100 N transducer. Low-temperature mechanical tests were conducted using a high/low-temperature electronic universal testing machine (Sinter, GWDW). Rectangular tensile specimens (40 mm × 10 mm × 2 mm) were tested at tensile and recovery rates of 50 mm min^-1^. Cylindrical compression specimens (12 mm diameter, 8 mm height) were tested at compression and recovery rates of 10 mm min^-1^.

**DMA measurements:** A TA DMA850 was used to study the dynamic mechanical properties of the eutectogels. Dynamic temperature scans (1 Hz, -30 to 80 °C) were conducted on cylindrical eutectogel samples (12 mm diameter, 3 mm height) using a compression fixture. The storage modulus, loss modulus, and loss tangent were determined across various temperatures.

**Conductivity measurements:** The resistance (R) of rectangular eutectogel specimens (40 mm × 10 mm × 2 mm) was measured using a TH2832 LCR digital bridge (Tonghui, China). The conductivity σ = L/RS, where L and S are the thickness and cross-sectional area of the sample, respectively.

Table S1 Proportions of different solutions in the reduction of HAuCl_4_·3H_2_O

|  | H_2_O (g) | EG (g) | urea (g) | ChCl (g) | 0.75 g  PVP  **+**  0.04 g  PSS  **+**  10 mg HAuCl_4_·3H_2_O |
| --- | --- | --- | --- | --- | --- |
| EG | - | 12.5 | - | - |  |
| EG + ChCl | - | 8 | - | 4.5 |  |
| EG + Urea | - | 11.15 | 1.35 | - |  |
| EG + Urea + ChCl | - | 7.44 | 0.9 | 4.16 |  |
| H_2_O | 12.5 | - | - | - |  |
| H_2_O + urea | 11.15 | - | 1.35 | - |  |
| H_2_O + urea+ ChCl | 7.44 | - | 0.9 | 4.16 |  |


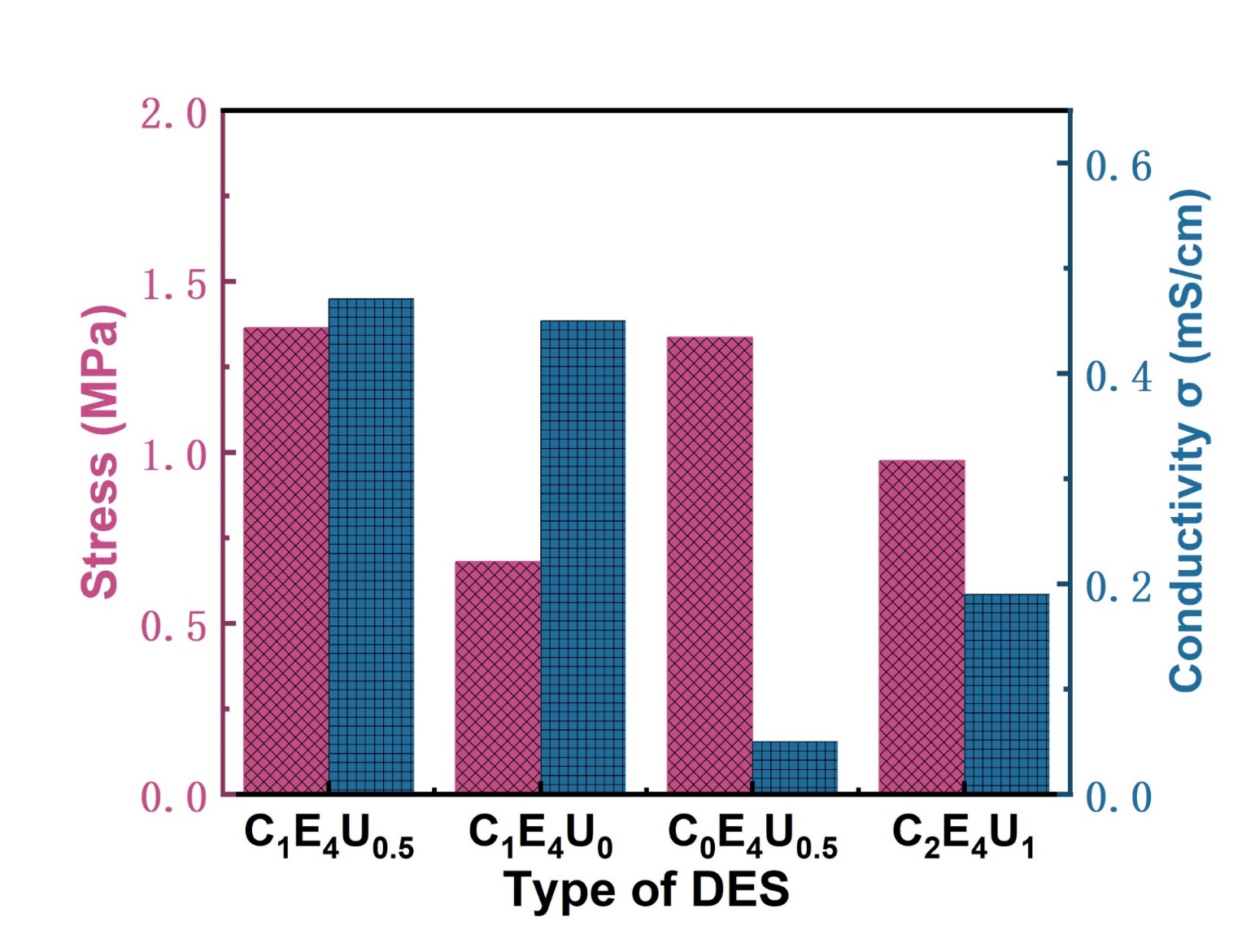


**Figure S1** Stress and electrical conductivity of eutectogels with varying CEU compositional ratios.


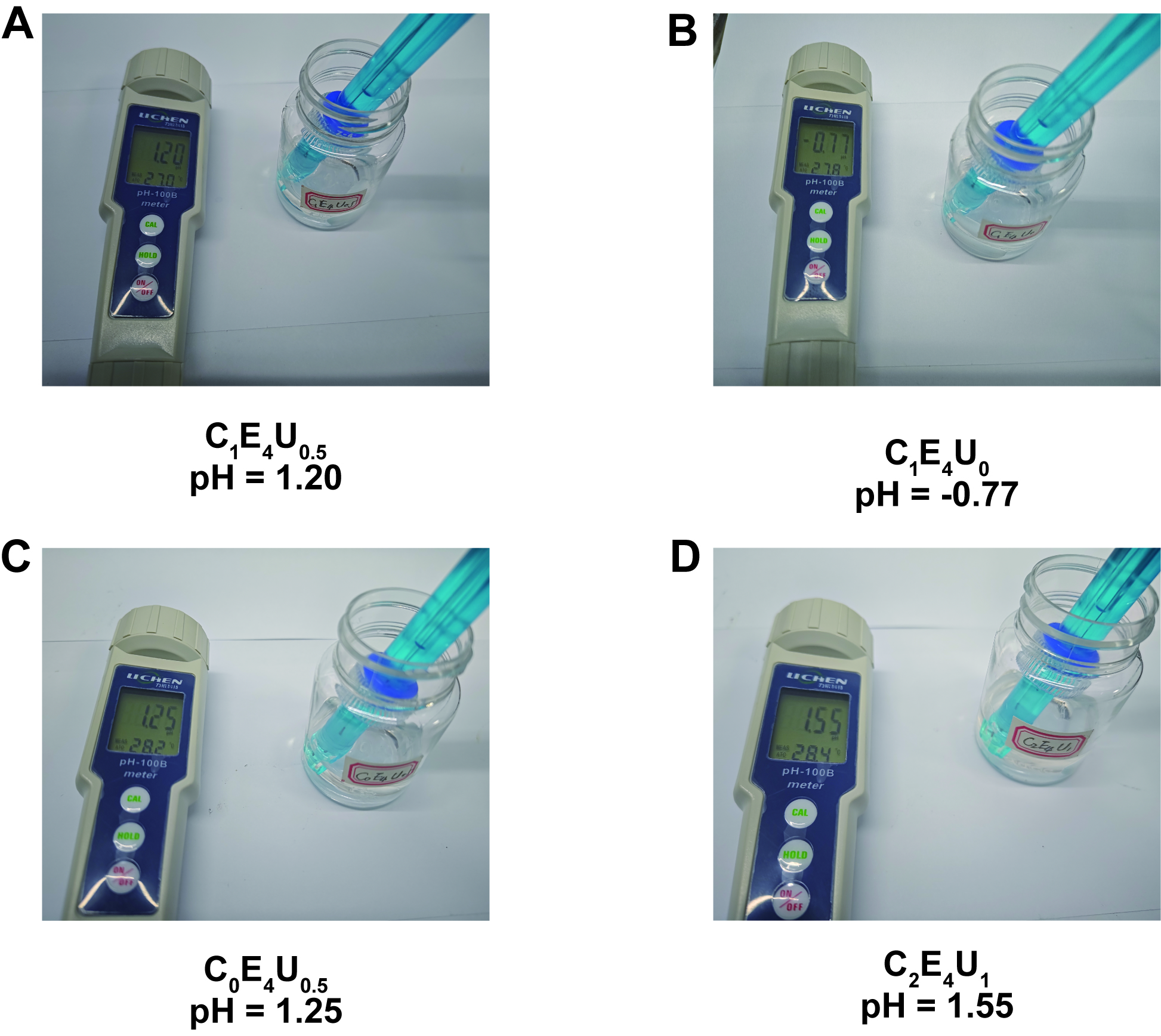


**Figure S2** Photographs showing the pH of PAA prepolymer solutions with different CEU compositions: (A) C_1_E_4_U_0.5_, (B) C_1_E_4_U_0_, (C) C_0_E_4_U_0.5_, and (D) C_2_E_4_U_1_.


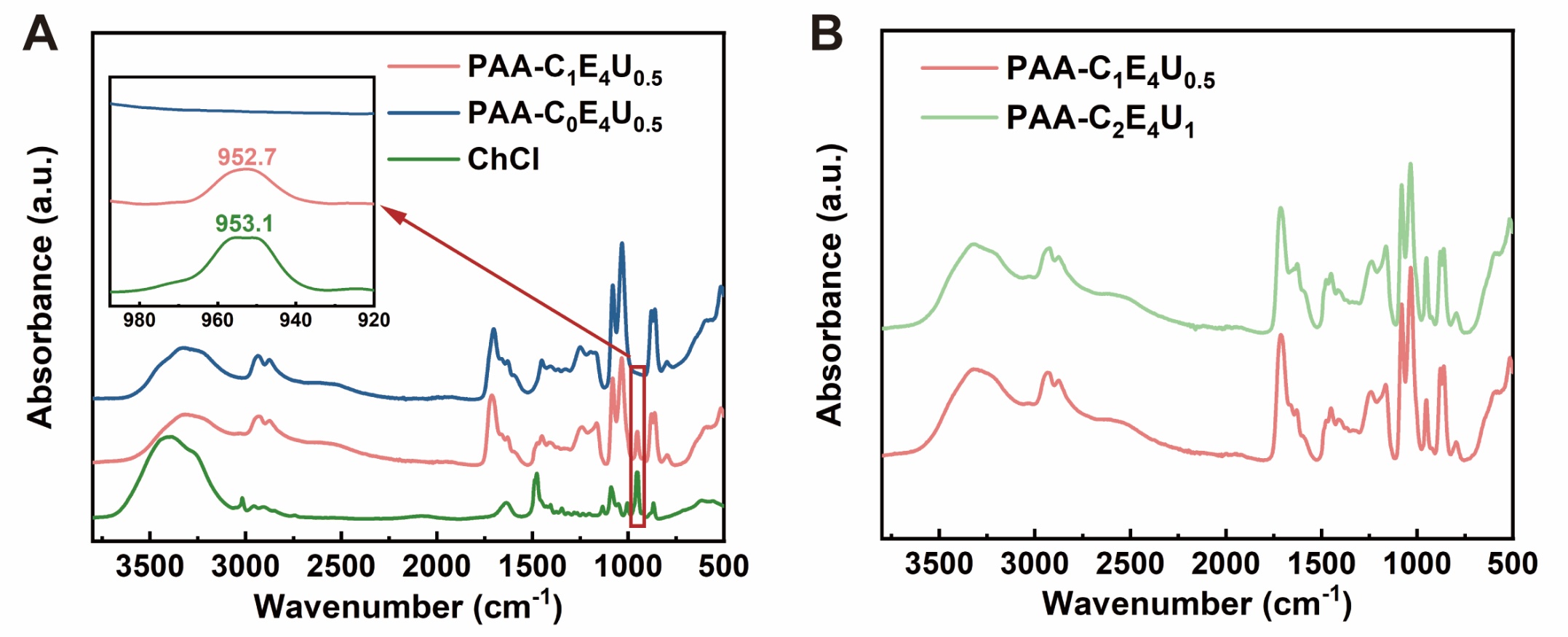


**Figure S3** FTIR spectra of ChCl and PAA eutectogels with varying CEU compositional ratios.


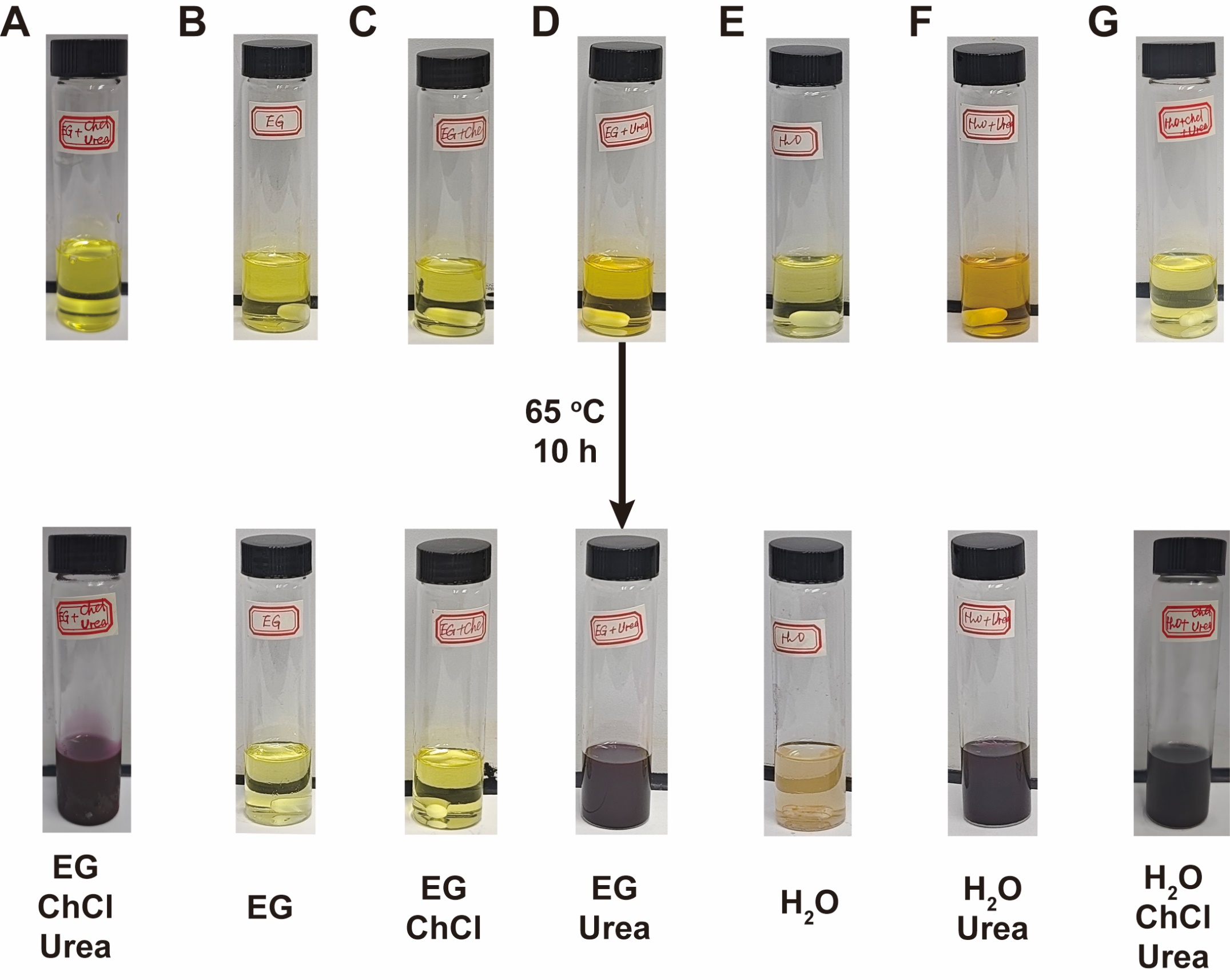


**Figure S4** Photographs showing the reduction of HAuCl_4_·3H_2_O in different solutions, before and after the reaction.


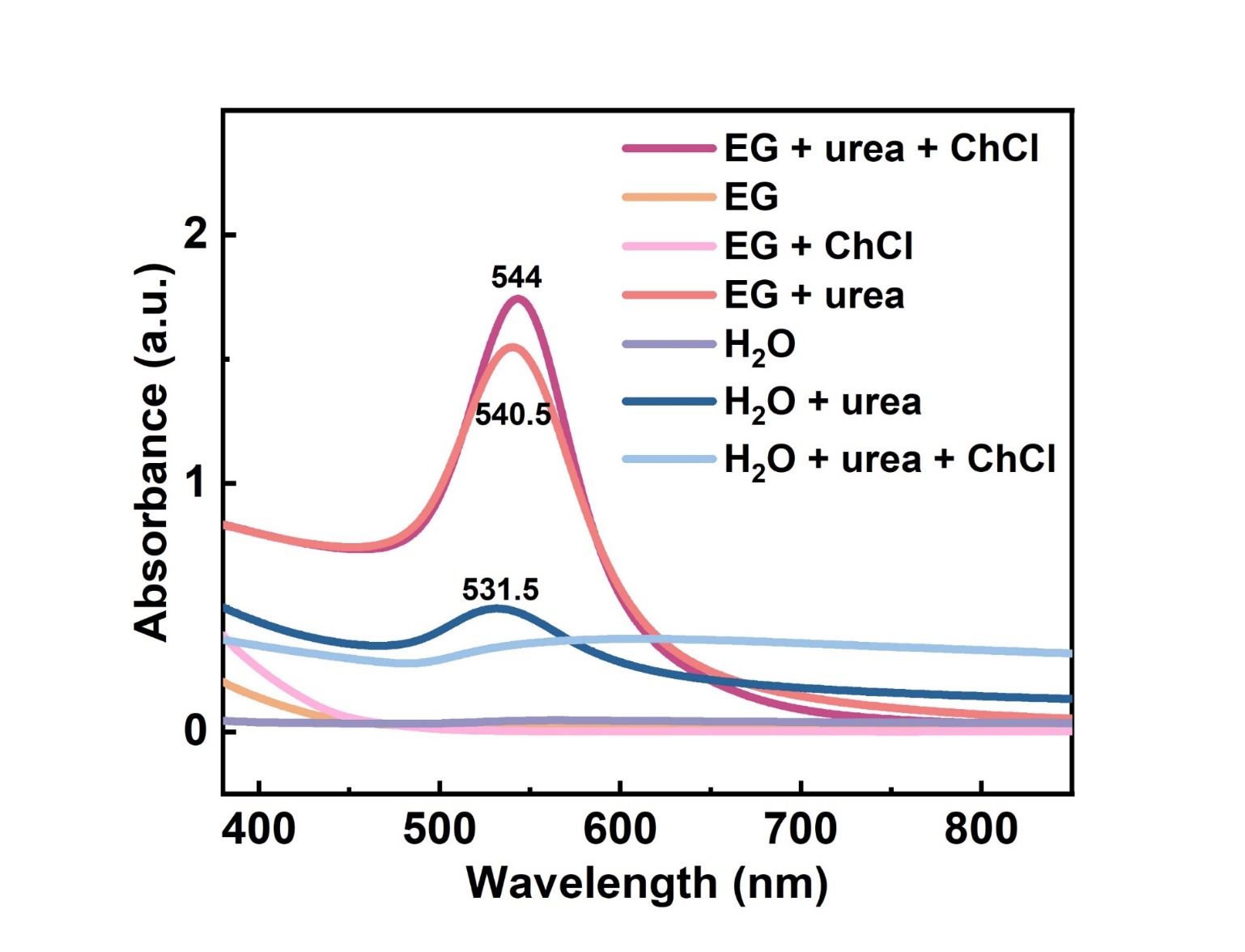


**Figure S5** UV-visible-NIR absorption spectra of various components during the reduction of HAuCl_4_·3H_2_O at 65°C for 10 h.


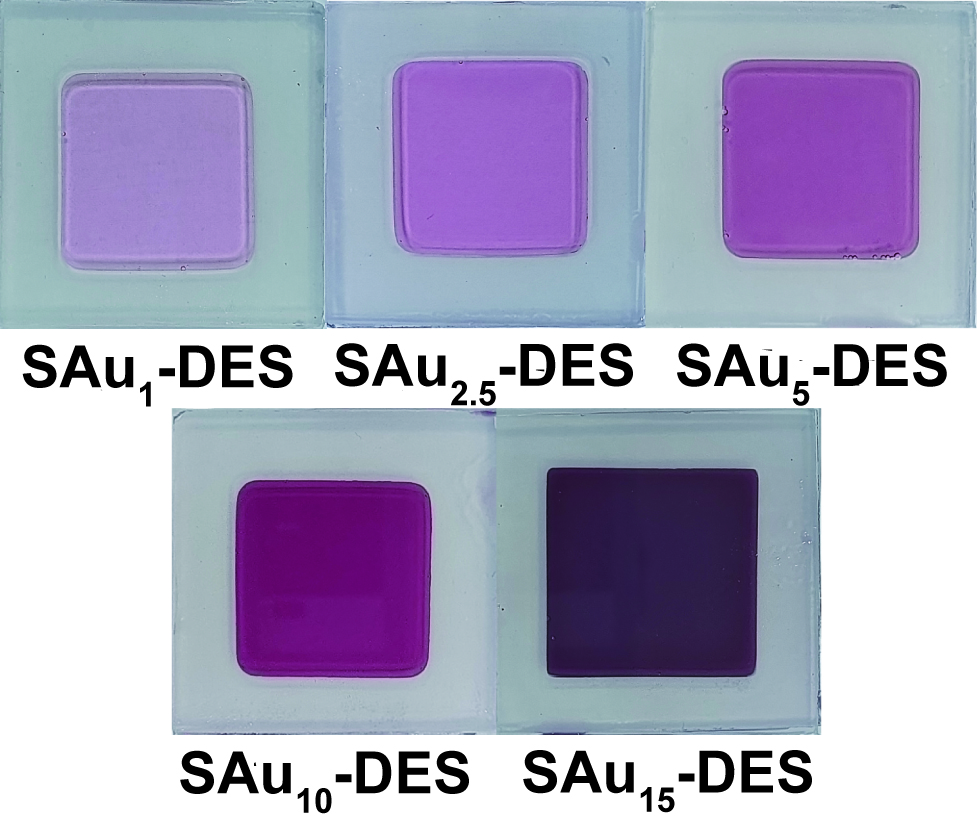


**Figure S6** Photographs showing the color variation of SAu-CEU solutions with varying AuNP concentrations.


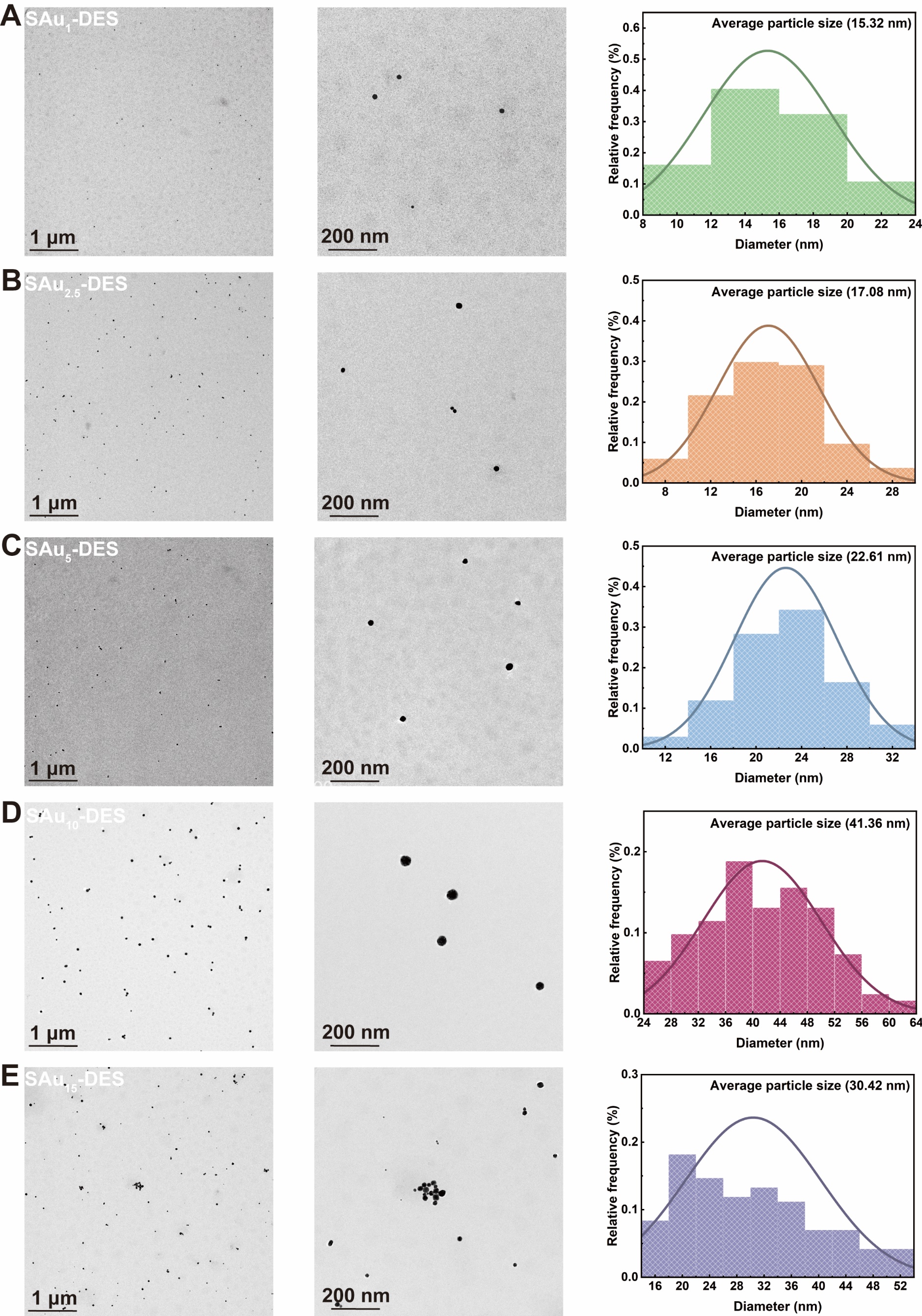


**Figure S7** SEM images of (A) SAu_1_-CEU, (B) SAu_2.5_-CEU, (C) SAu_5_-CEU, (D) SAu_10_-CEU, and (E) SAu_15_-CEU solutions after drying, along with the corresponding particle-size distribution statistics.


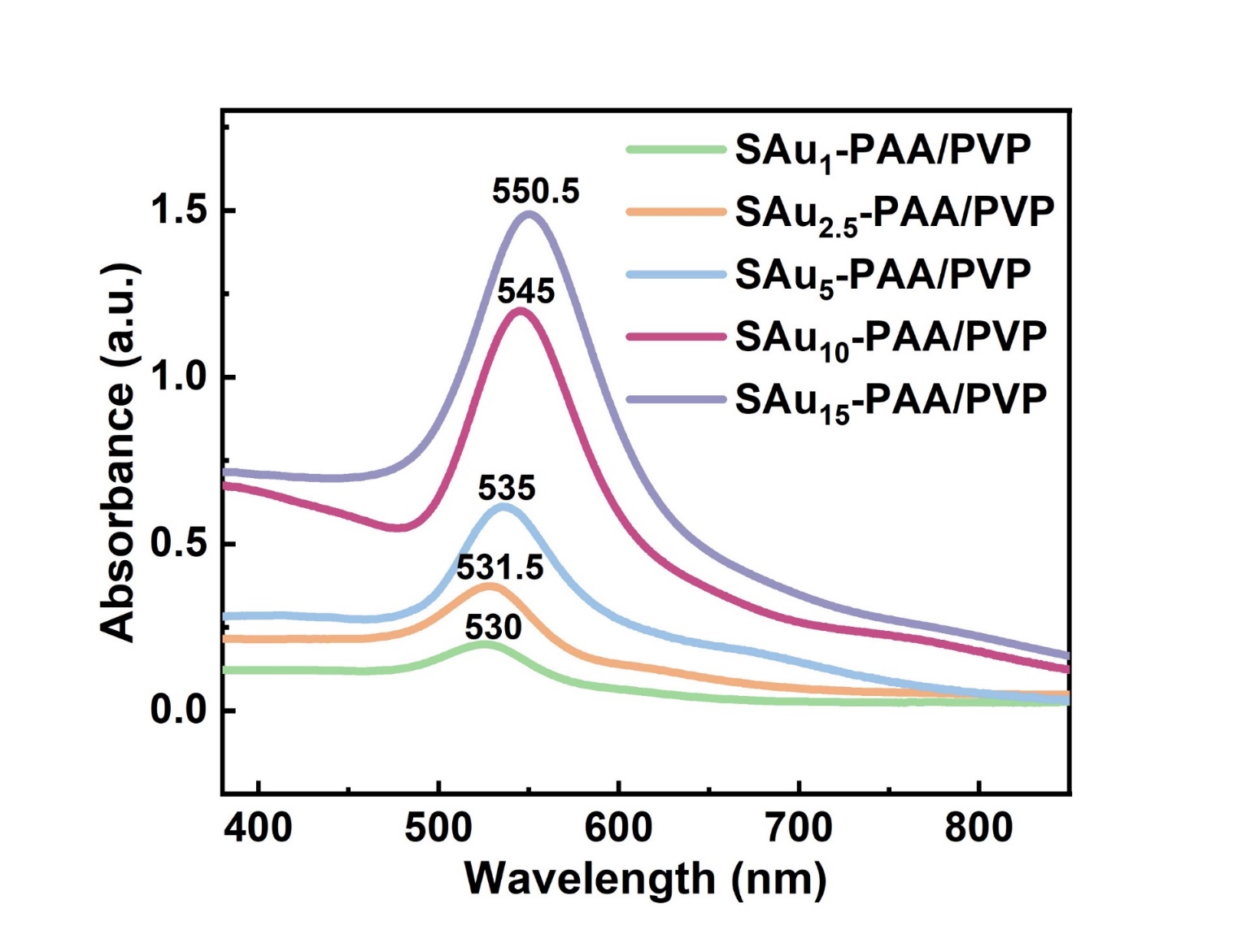


**Figure S8** UV-visible-NIR absorption spectra of SAu-CEU solutions with varying AuNP concentrations.


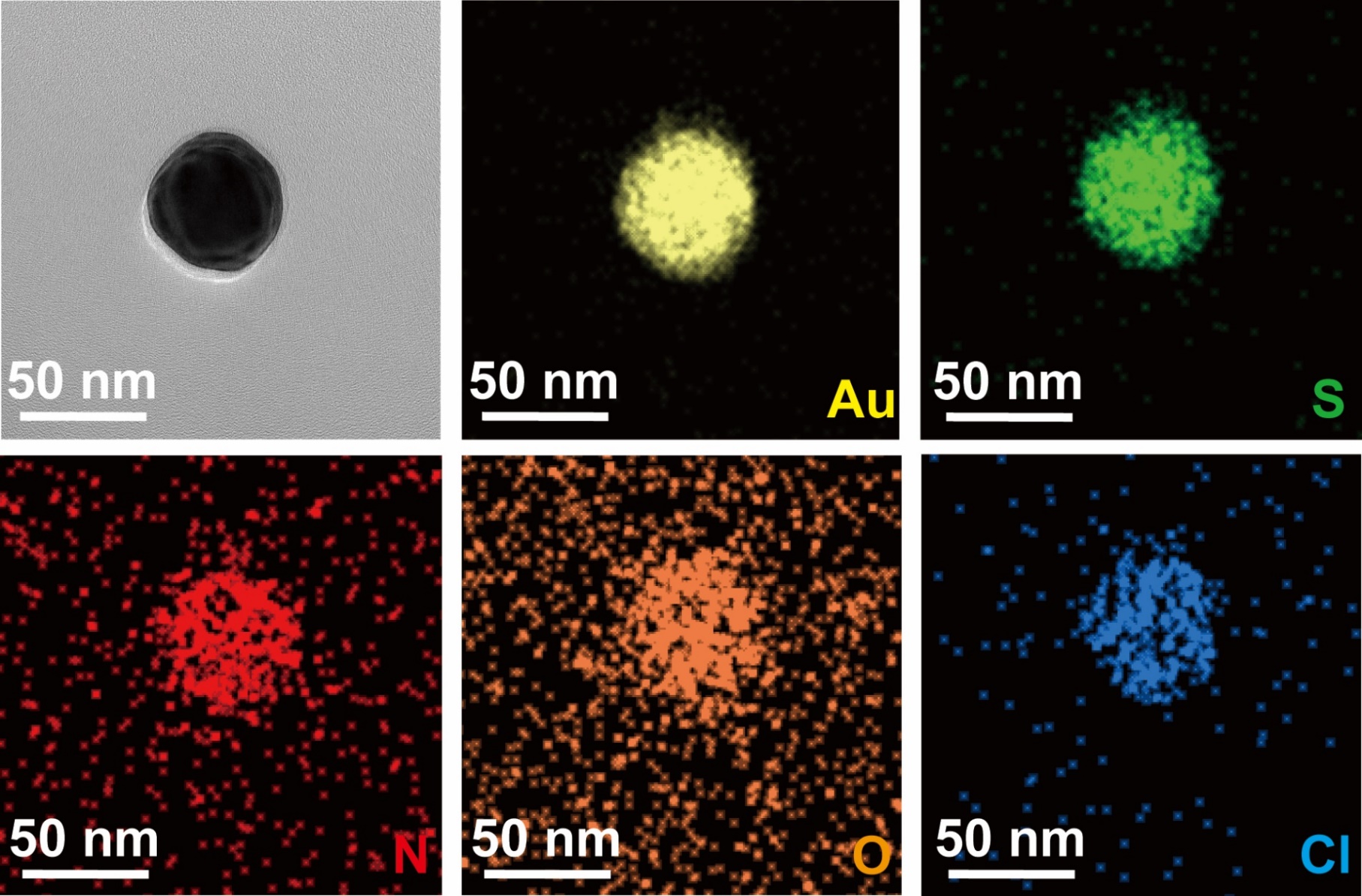


**Figure S9** SEM image of an individual PSS-modified AuNP with its corresponding EDS elemental maps.


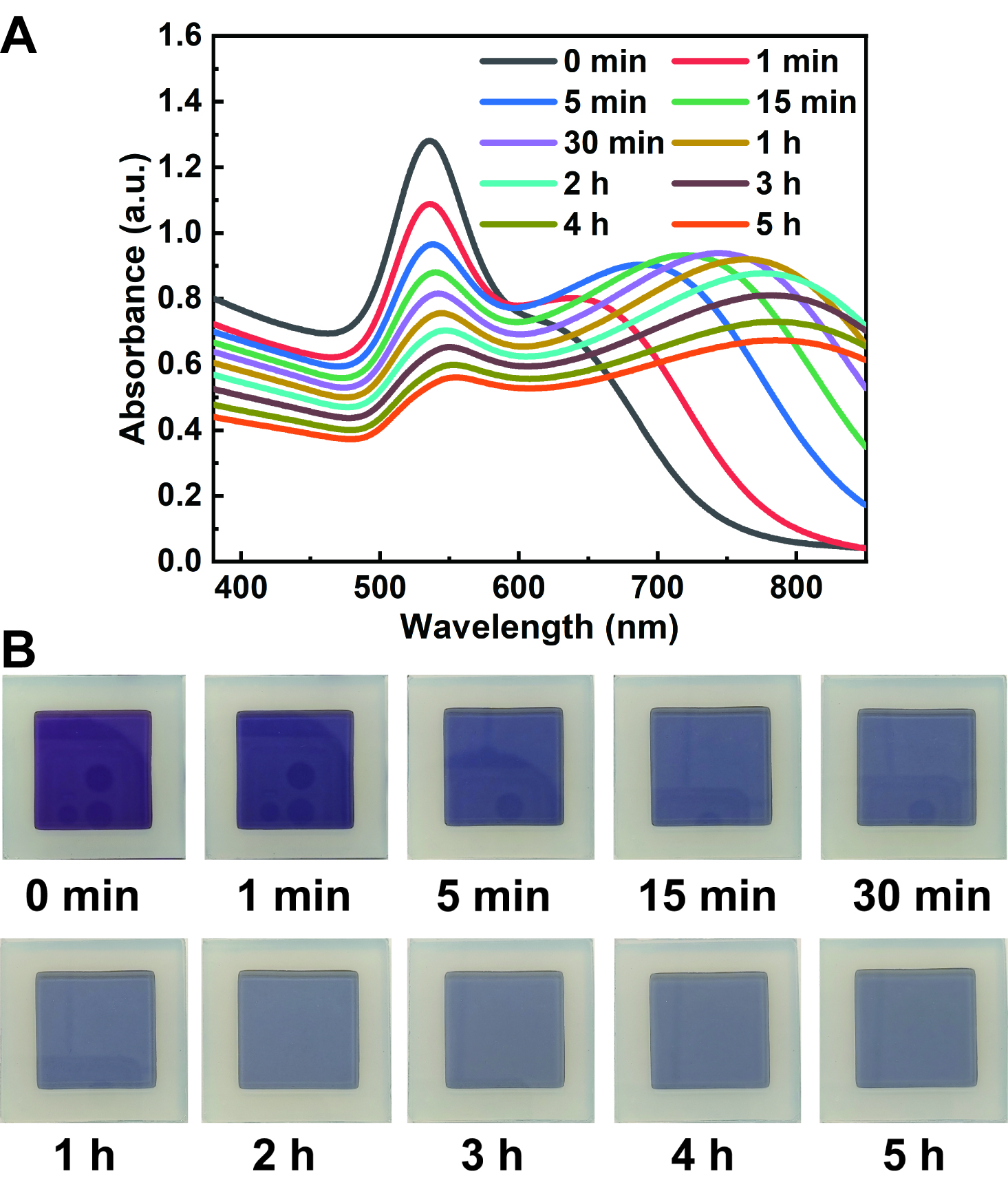


**Figure S10** (A) UV-visible-NIR absorption spectra and (B) photographs showing the color variations of unmodified AuNPs mixed with PAA prepolymer solutions over time.


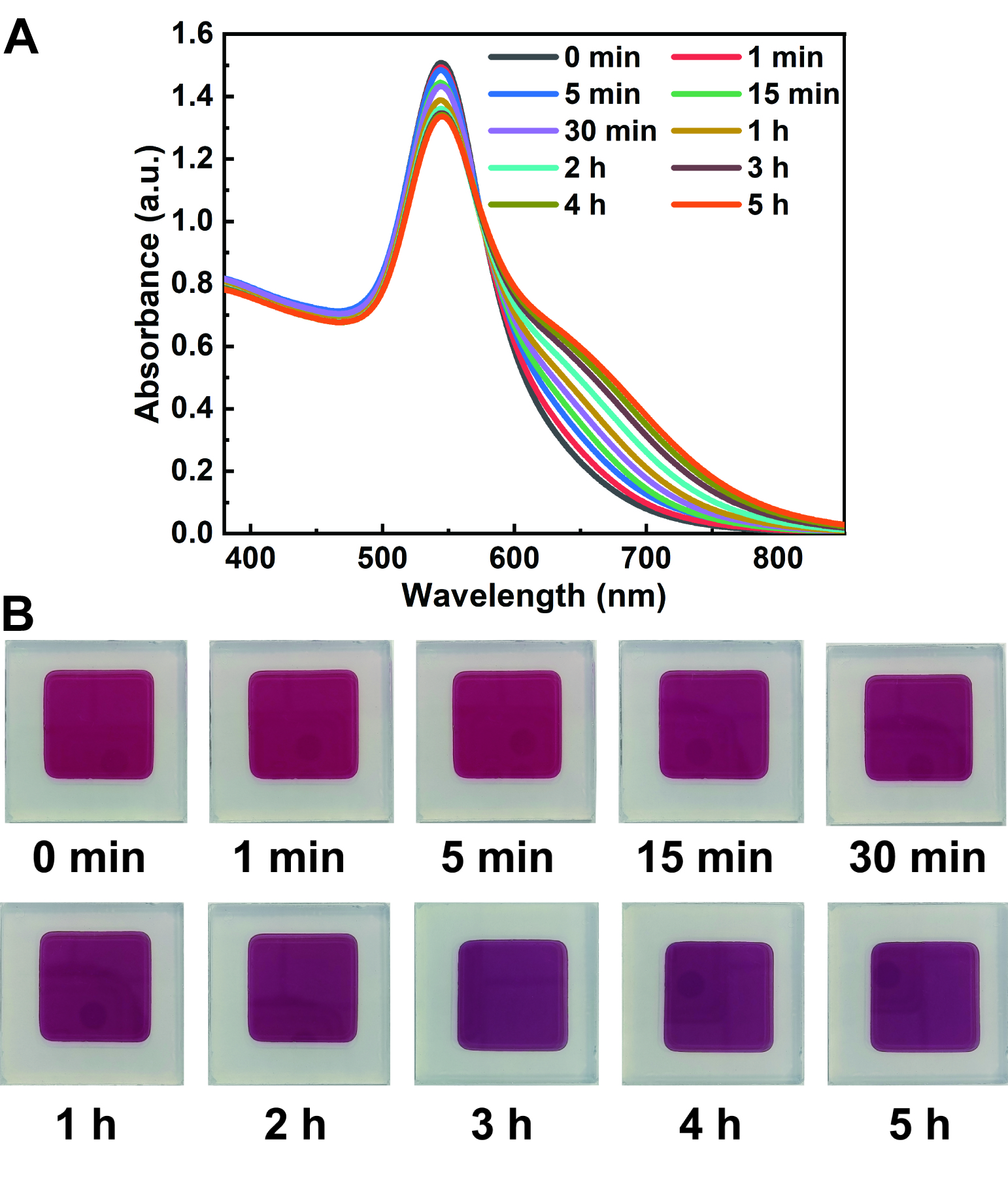


**Figure S11** (A) UV-visible-NIR absorption spectra and (B) photographs showing the color variations of SAu-CEU mixed with PAA eutectogel prepolymer solutions over time.


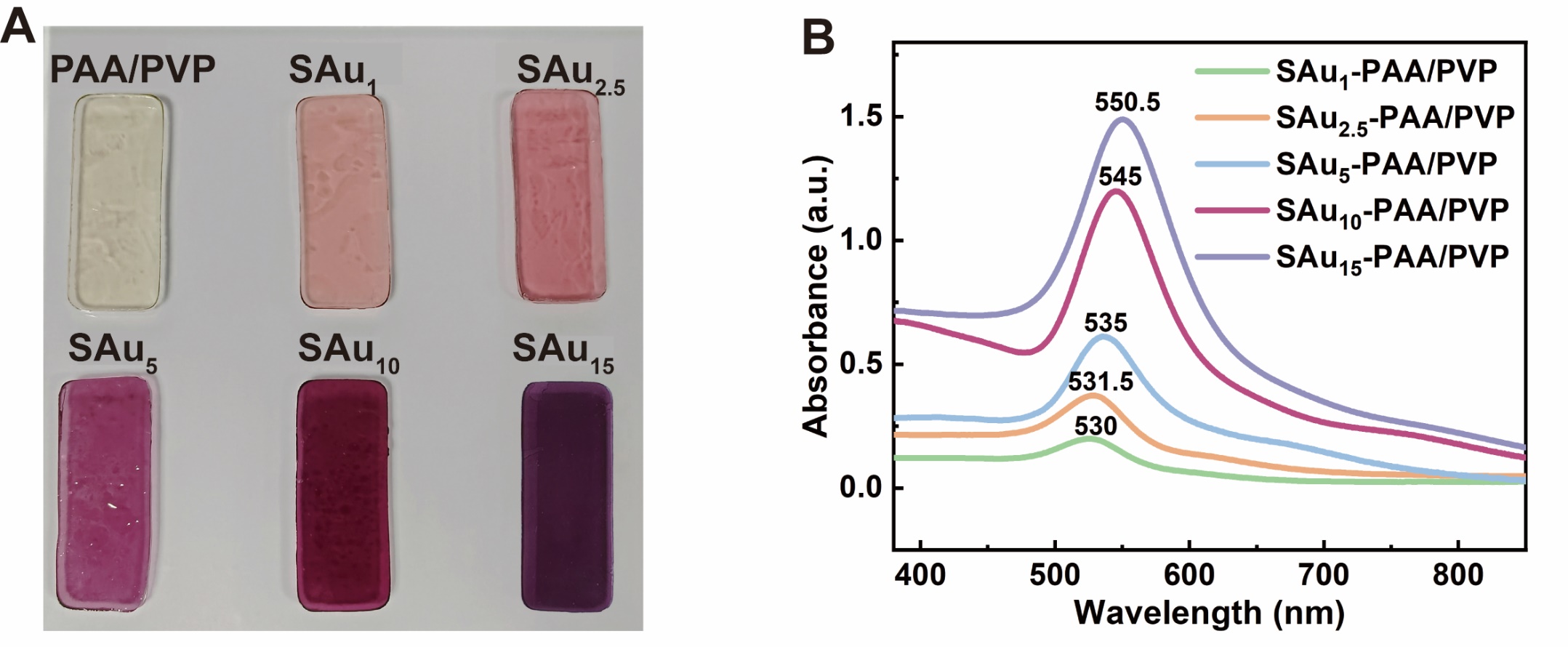


**Figure S12** (A) Photograph and (B) UV-visible-NIR absorption spectra of SAu-PAA/PVP eutectogels with varying AuNP concentrations.


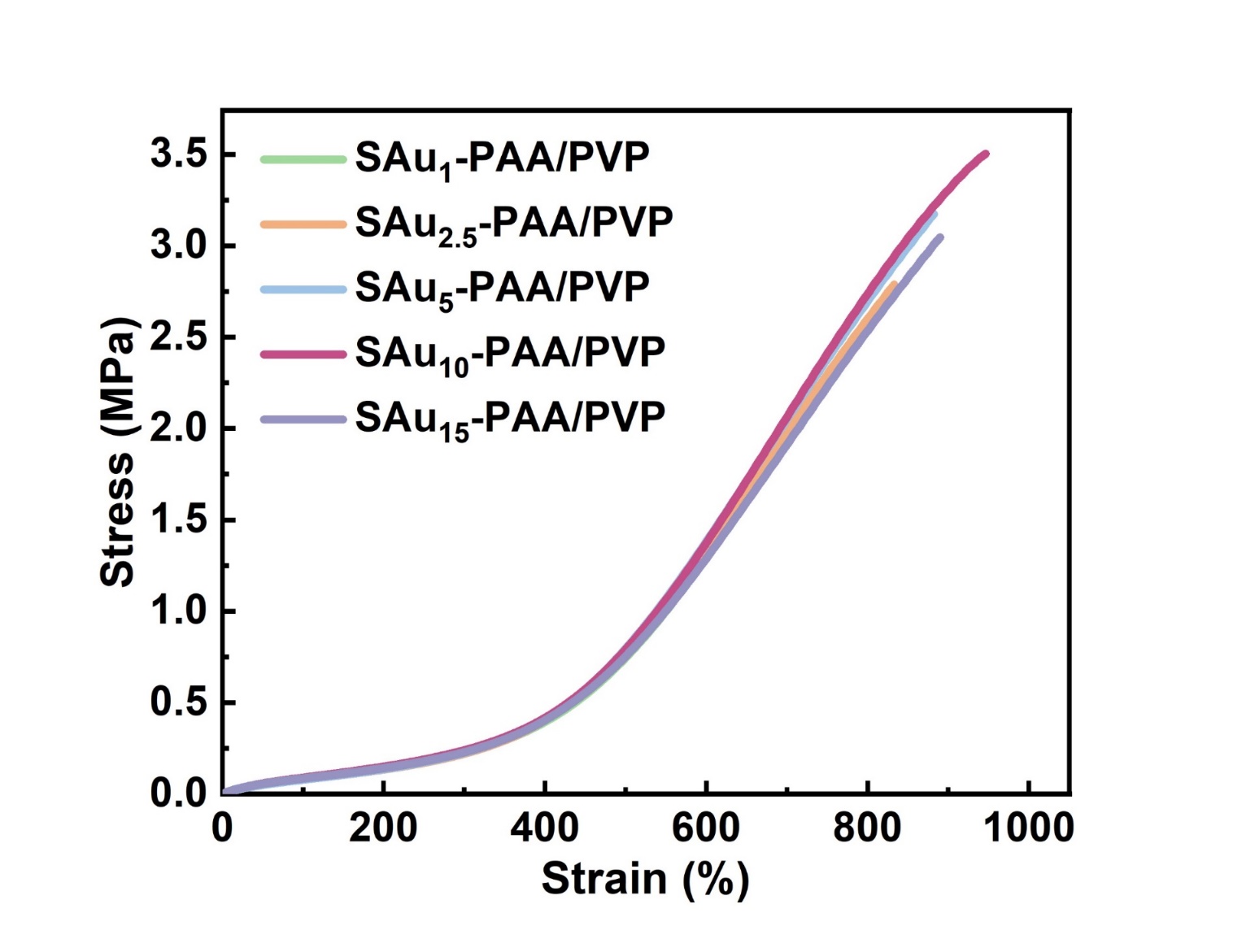


**Figure S13** Tensile stress-strain curves of SAu-PAA/PVP eutectogels with varying AuNP concentrations.


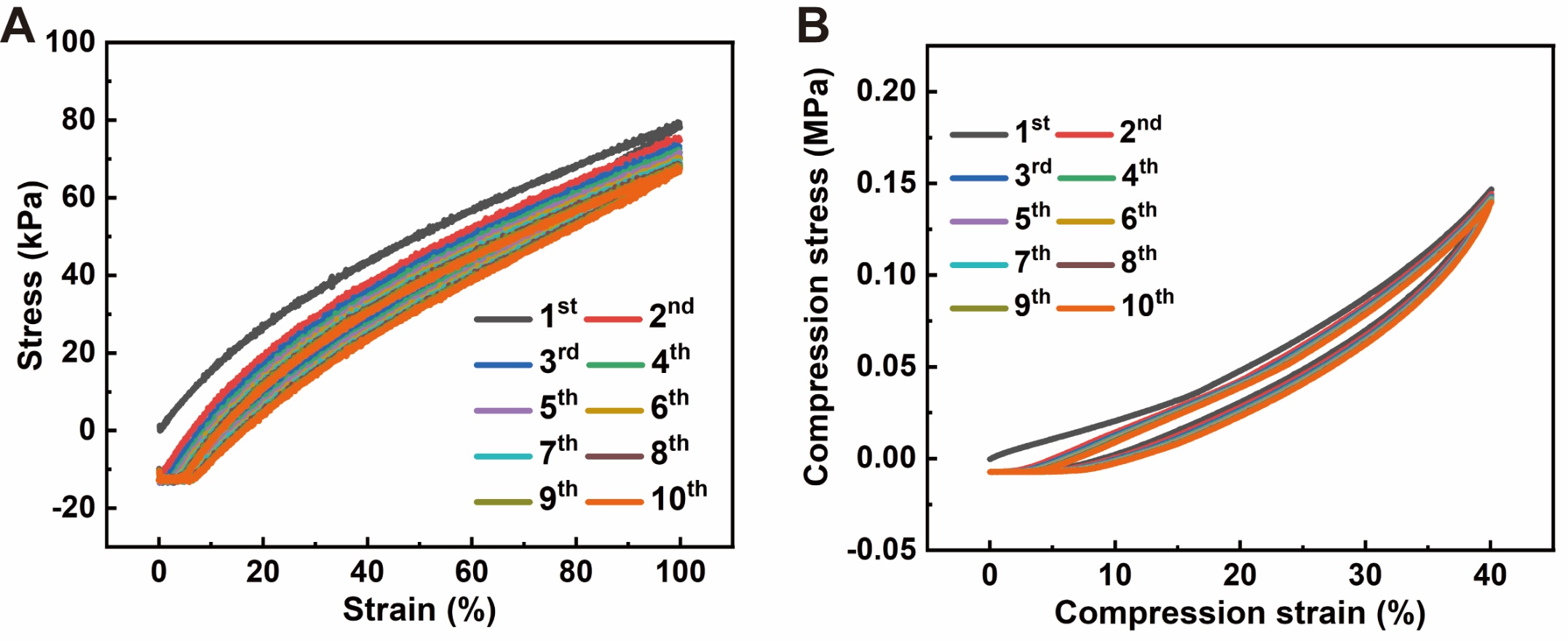


**Figure S14** (A) Loading-unloading cycles at 100% tensile strain and (B) compression-relaxation cycles at 40% compressive strain of a PAA/PVP eutectogel.


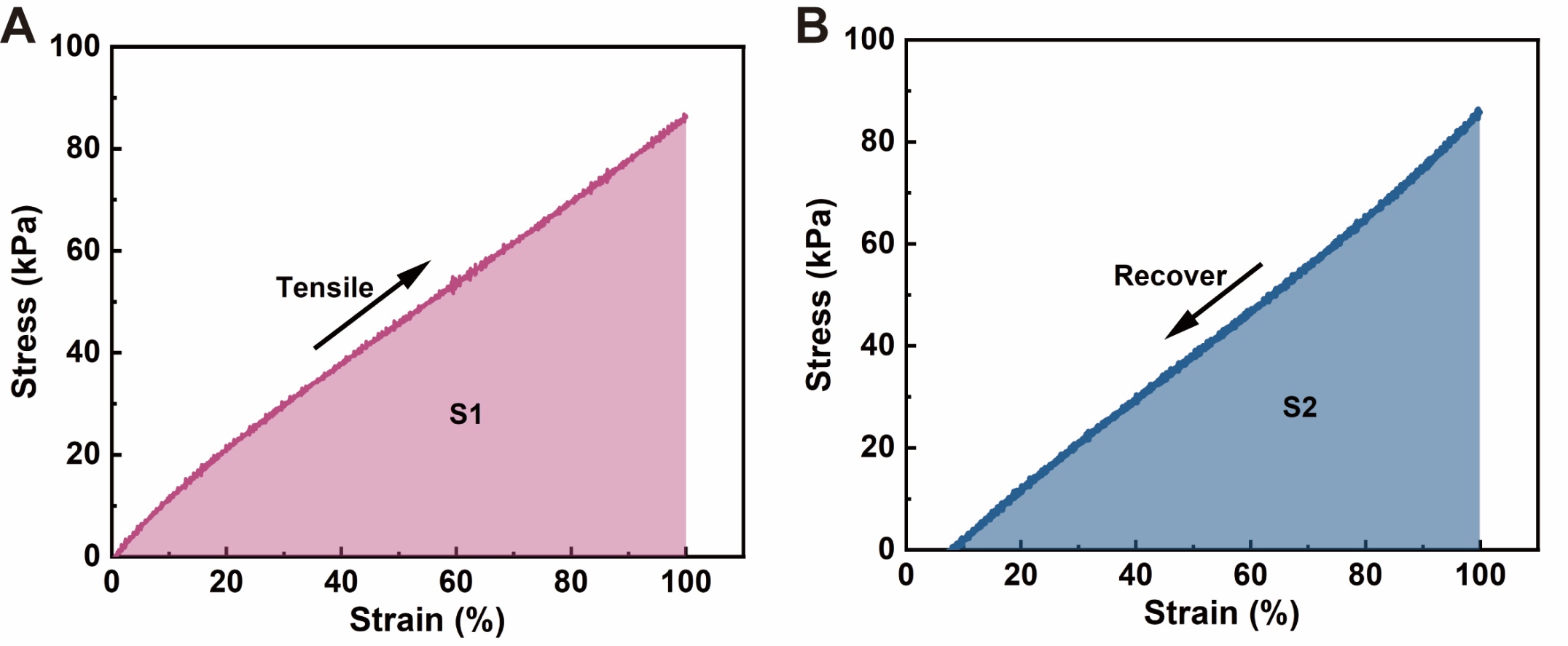


**Figure S15** (A) The 100% tensile curve and (B) recovery curve of the SAu-PAA/PVP eutectogel, along with the corresponding input energy (S1) and output energy (S2). The resilience (%) is calculated as S2/S1 * 100%.

**Table S2** Comparison of the Mechanical properties of the eutectogel developed in this work with other eutectogels reported in the literature.

| Polymer network structure | Strength at break  (MPa) | Elongation at break  (%) | Resilience  (%) | Ref. |
| --- | --- | --- | --- | --- |
| PAA/ cellulose nanocrystals | 0.66 | 1700 | 48.1 | 50 |
| PAA/cellulose | 0.87 | 887 | 25.4 | 51 |
| PVA/gelatin/cellulose | 1.25 | 1400 | 80.3 | 52 |
| PAA/ resorcinol-formaldehyde resin | 0.90 | 230 | 44.6 | 53 |
| PAA/ hydroxypropyl cellulose | 0.135 | 200 | 98.1 | 54 |
| PAA | 0.13 | 482.3 | 79.0 | 55 |
| Poly(N-hydroxymethyl acrylamide-co-hydroxyethyl methacrylate) | 0.935 | 648 | 63.7 | 56 |
| PAA/PVP | 3.50 | 946 | 85.3 | This work |

**
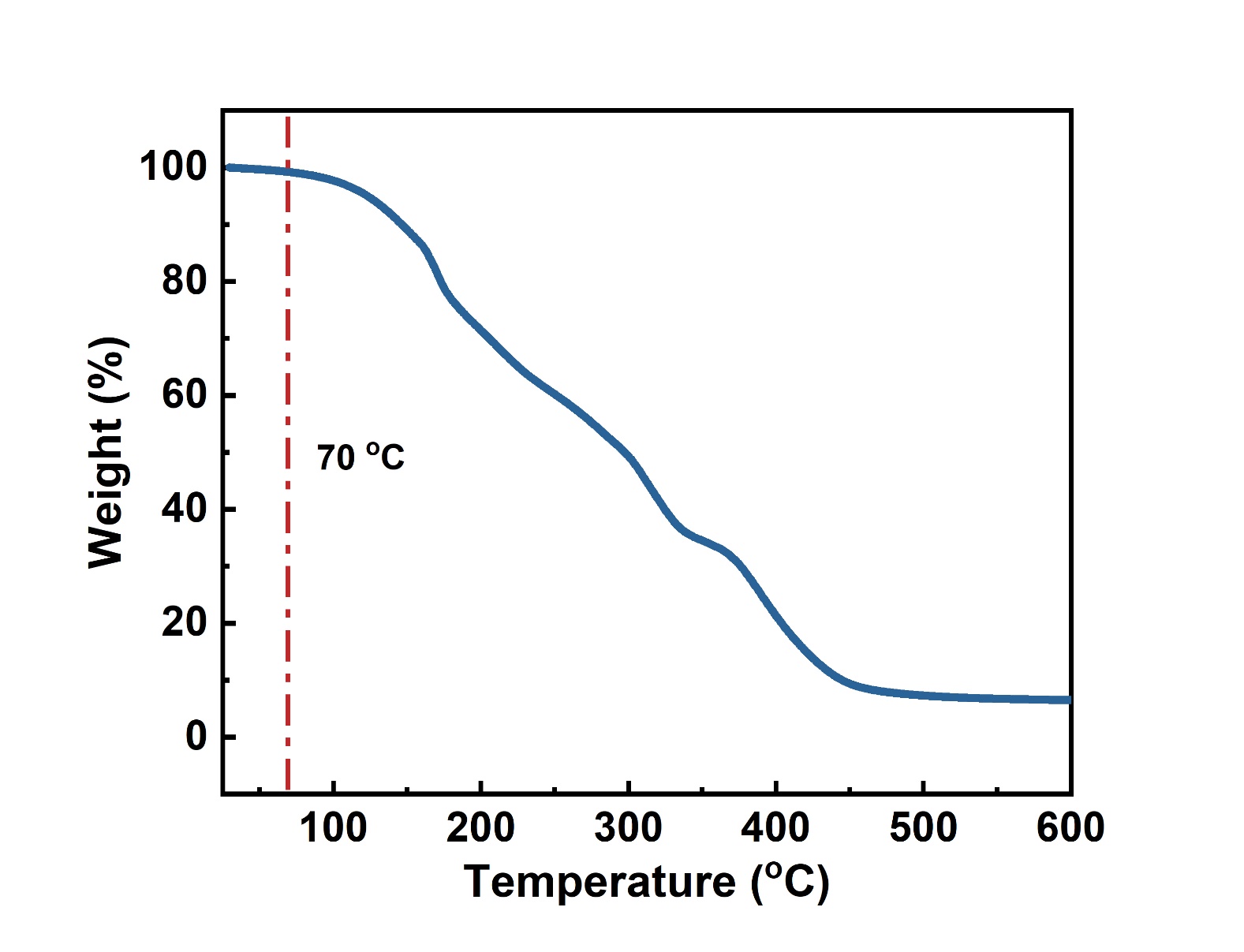
**

**Figure S16** TGA tests of SAu-PAA/PVP eutectogel from 25 to 600 ℃.


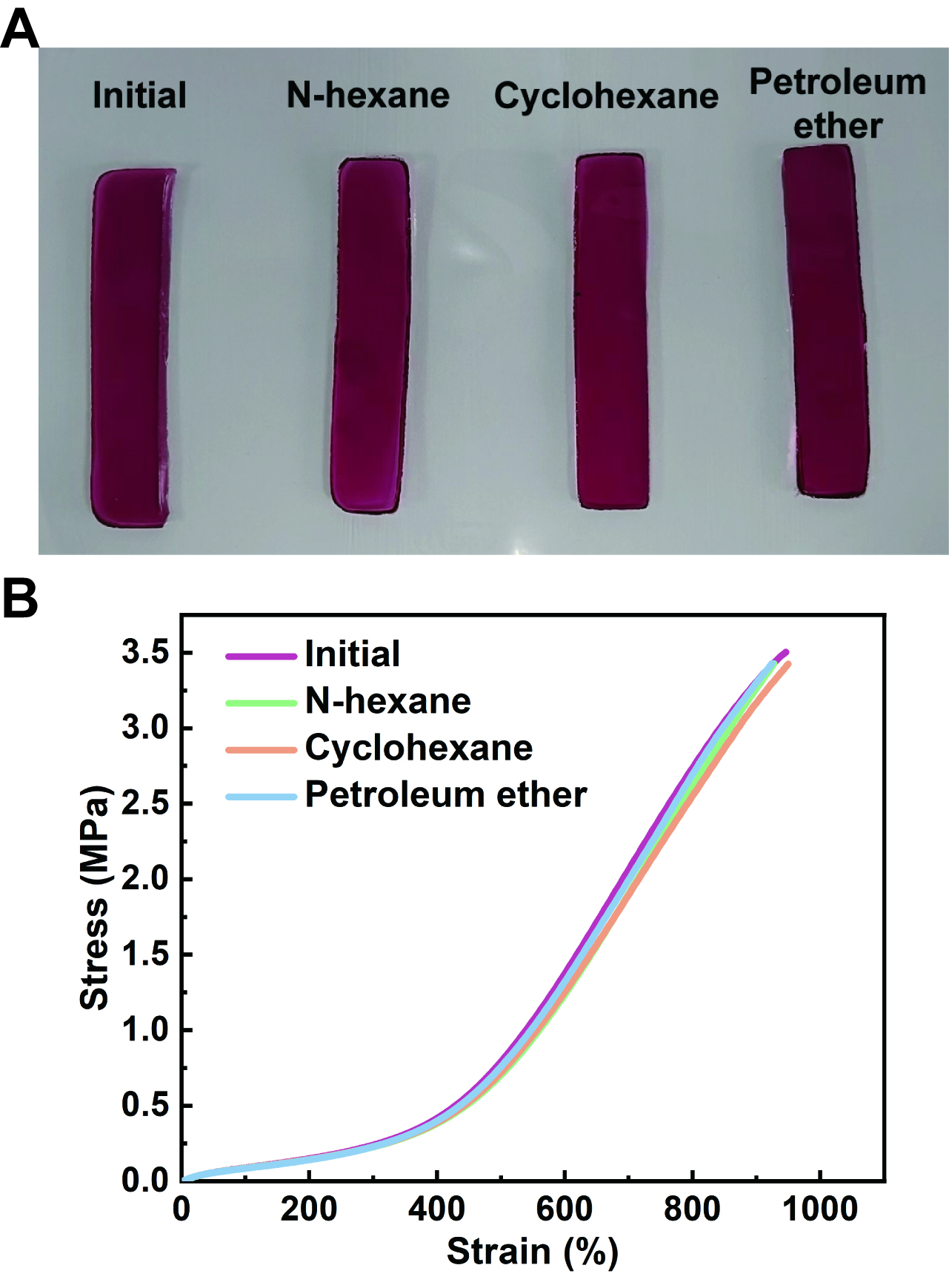


**Figure S17** (A) Photograph and (B) tensile stress-strain curves of SAu-PAA/PVP eutectogel after immersion in nonpolar solvents (N-hexane, cyclohexane, and petroleum ether) for 24 h.


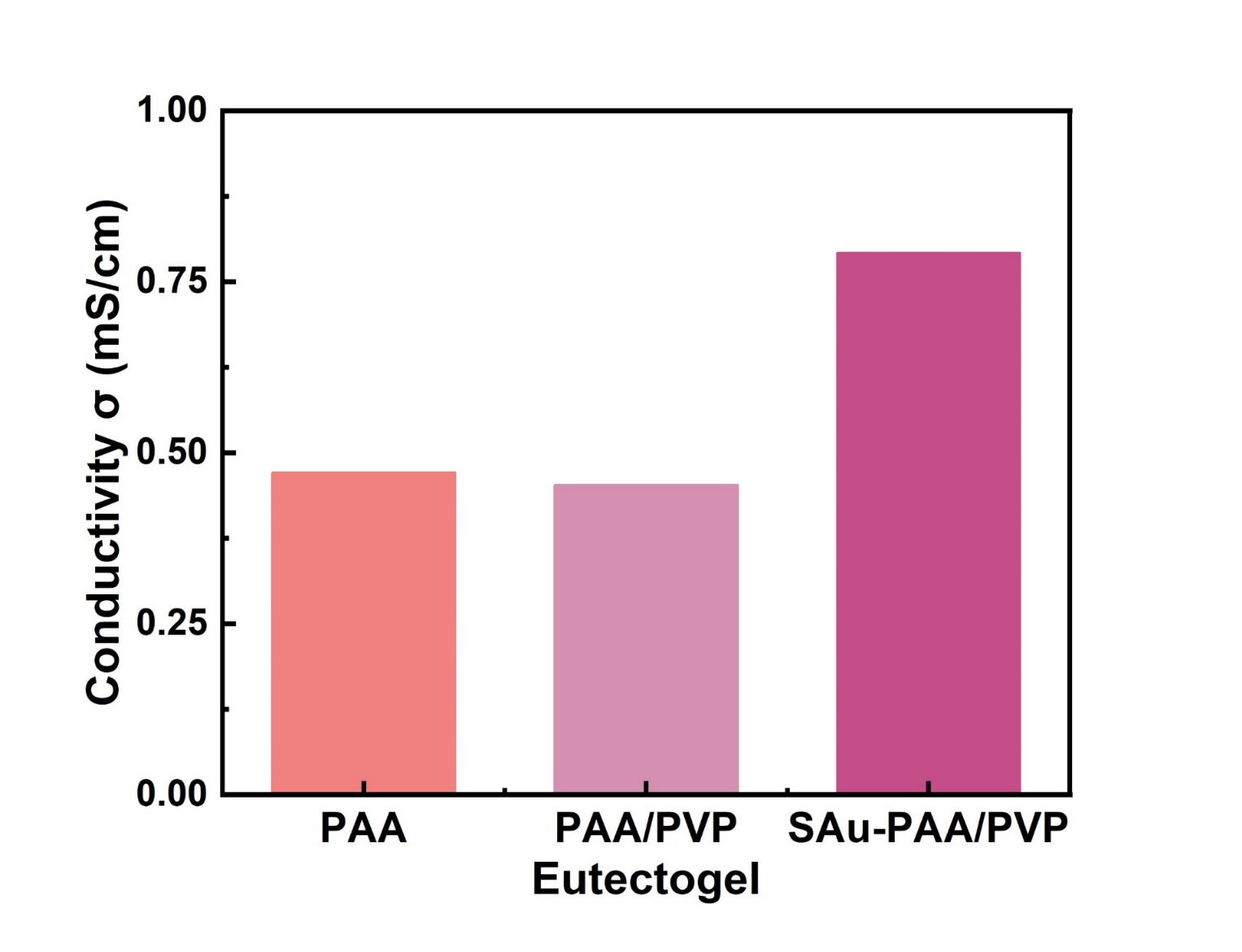


**Figure S18** Conductivity of different eutectogels.


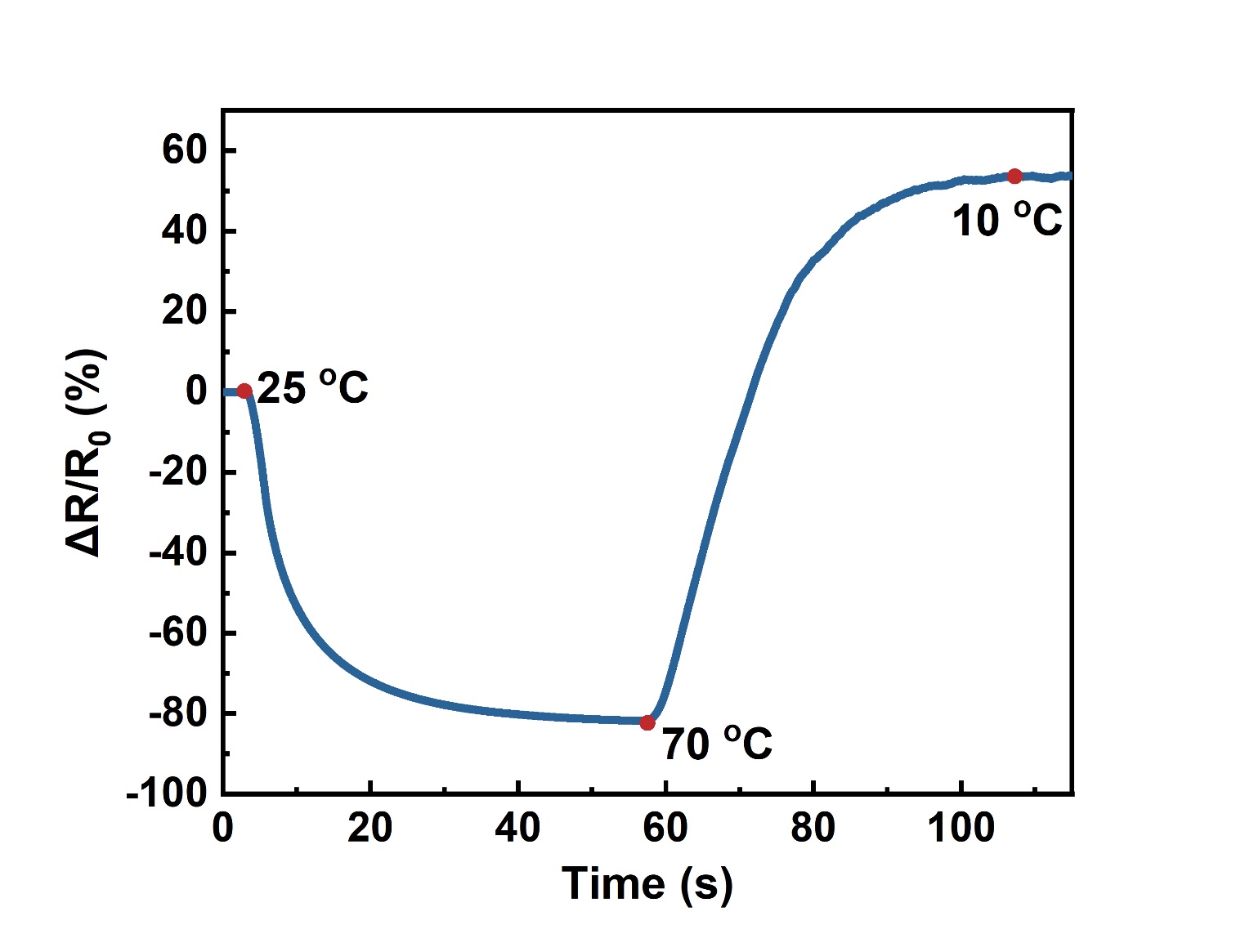


**Figure S19** ΔR/R_0_ of the SAu-PAA/PVP eutectogel under varying temperature environment.


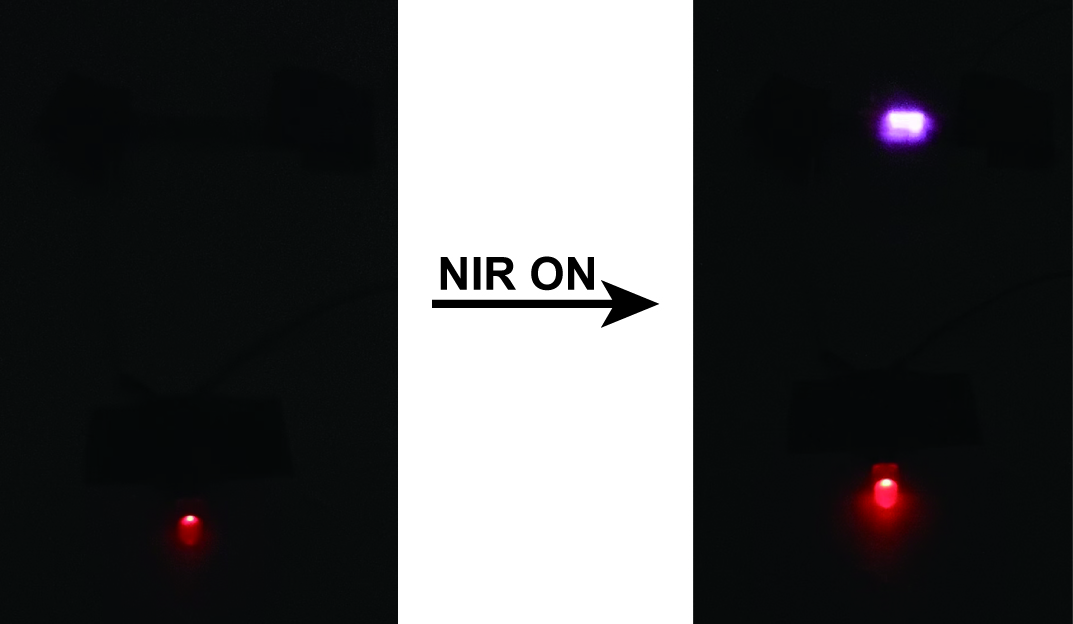


**Figure S20** Photographs of LEDs connected to an SAu-PAA/PVP eutectogel under 808 nm NIR light illumination, visualizing the resistance change.


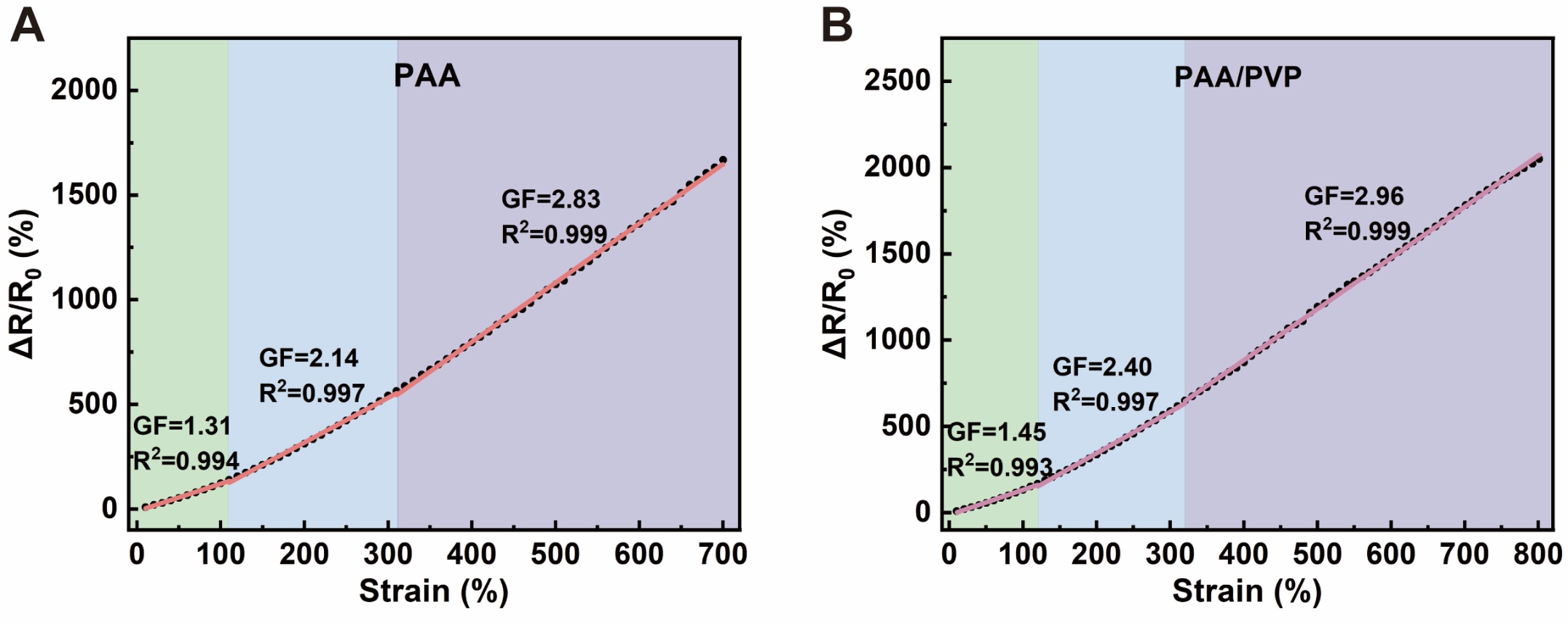


**Figure S21** Relative resistance changes of (A) PAA and (B) PAA/PVP eutectogels as functions of strain.


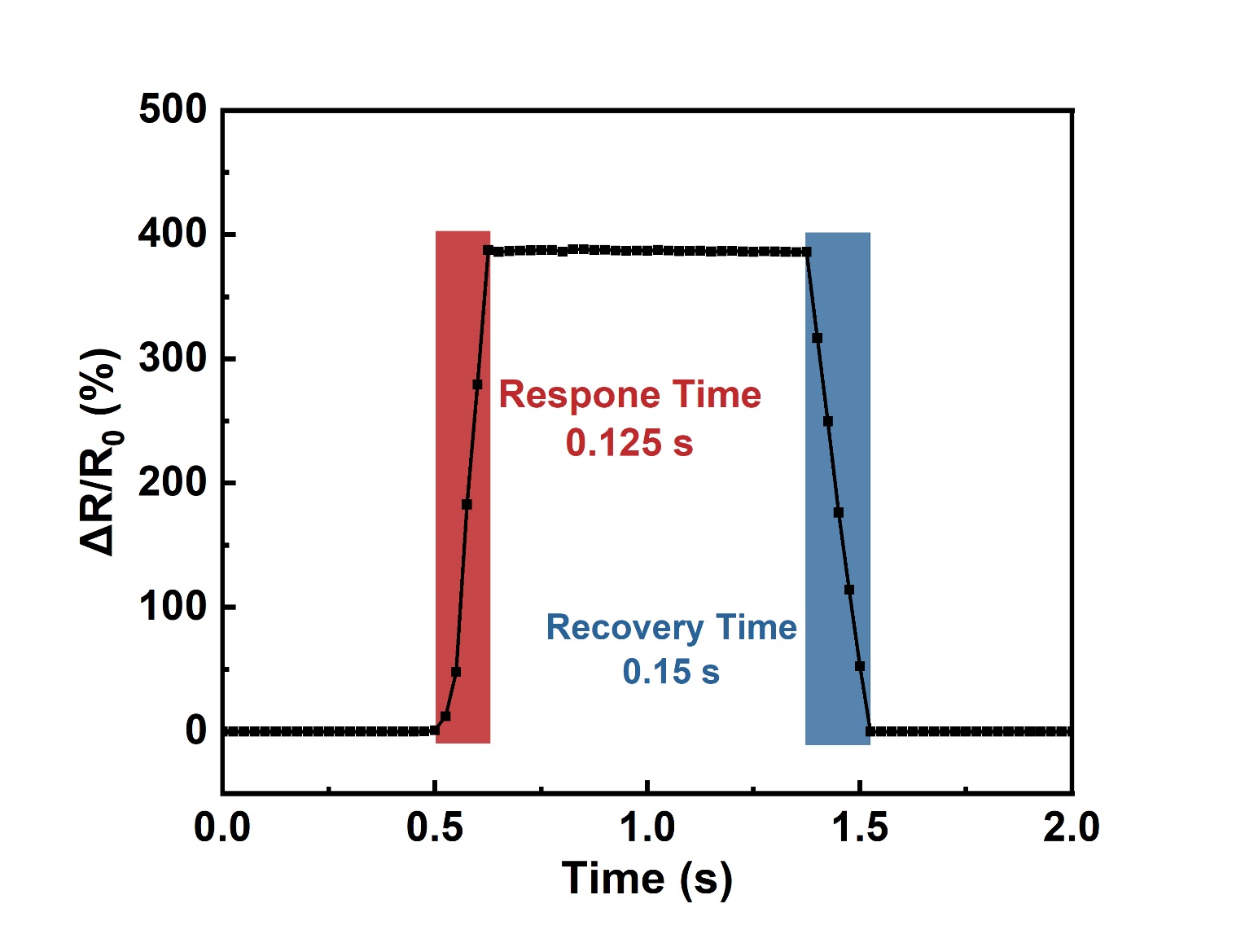


**Figure S22** Response/recovery time of an SAu-PAA/PVP eutectogel-based strain sensor.


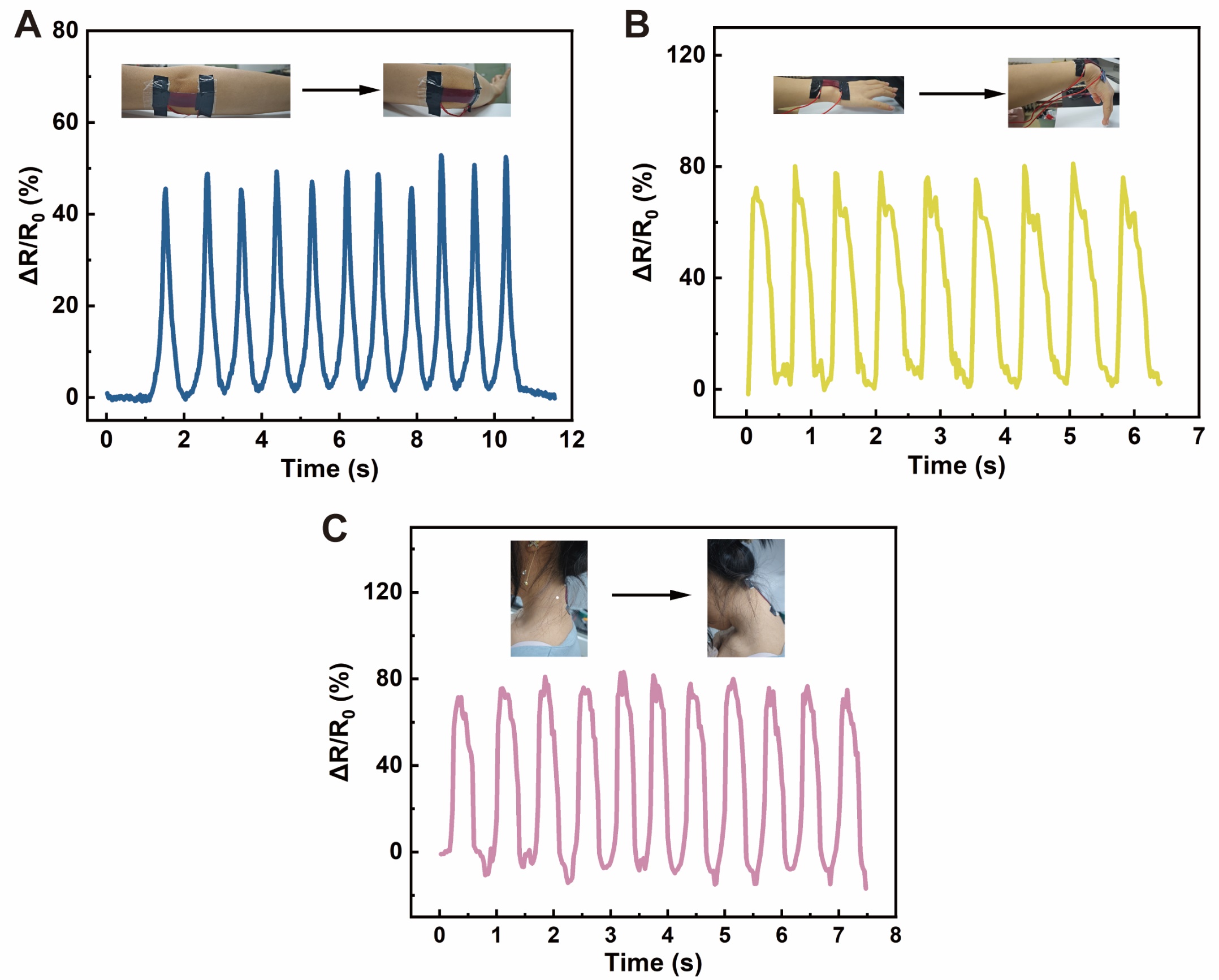


**Figure S23** Relative resistance change curves recorded when the sensor was placed on different body parts: (A) elbow, (B) wrist, and (C) neck.


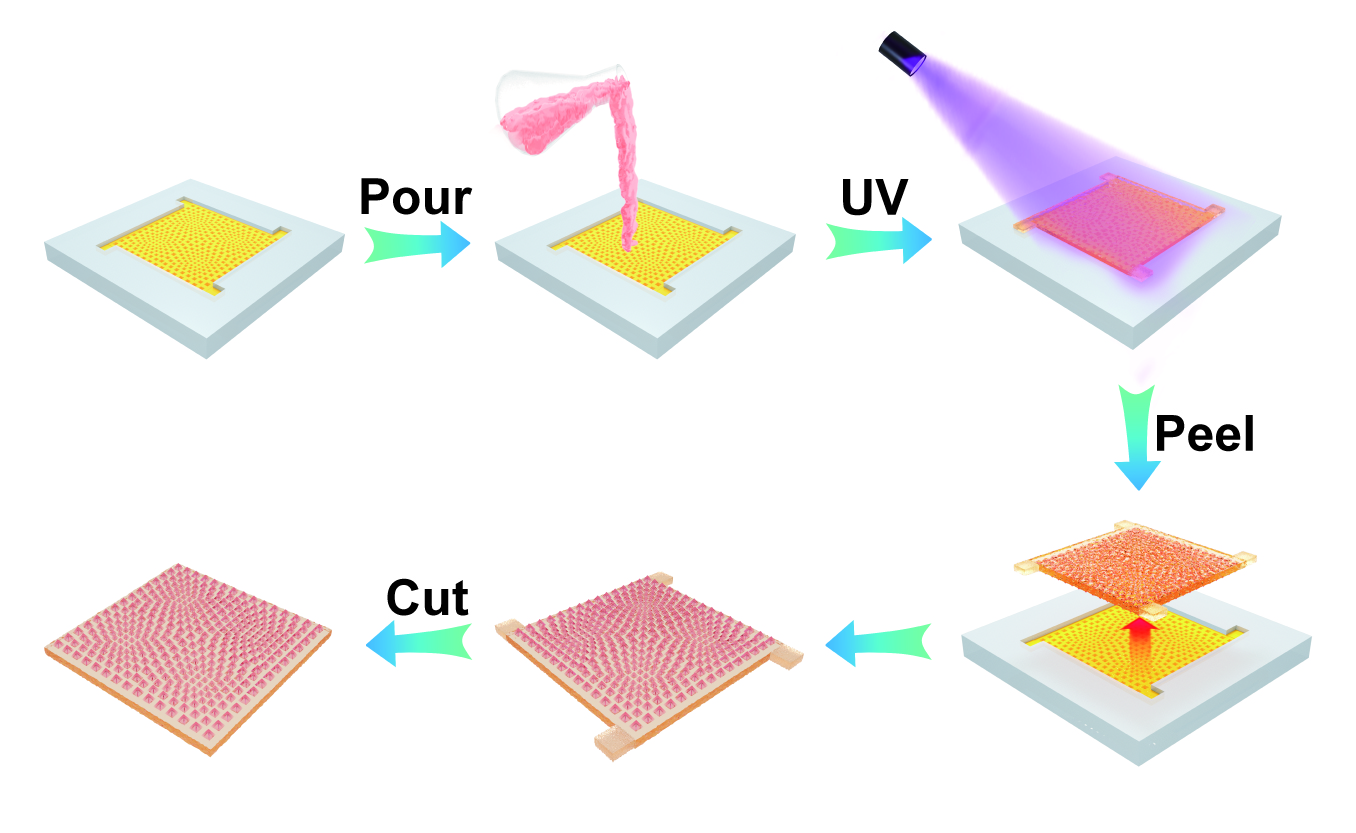


**Figure S24** Fabrication of SAu-PAA/PVP eutectogel microstructured ionic dielectric layers.


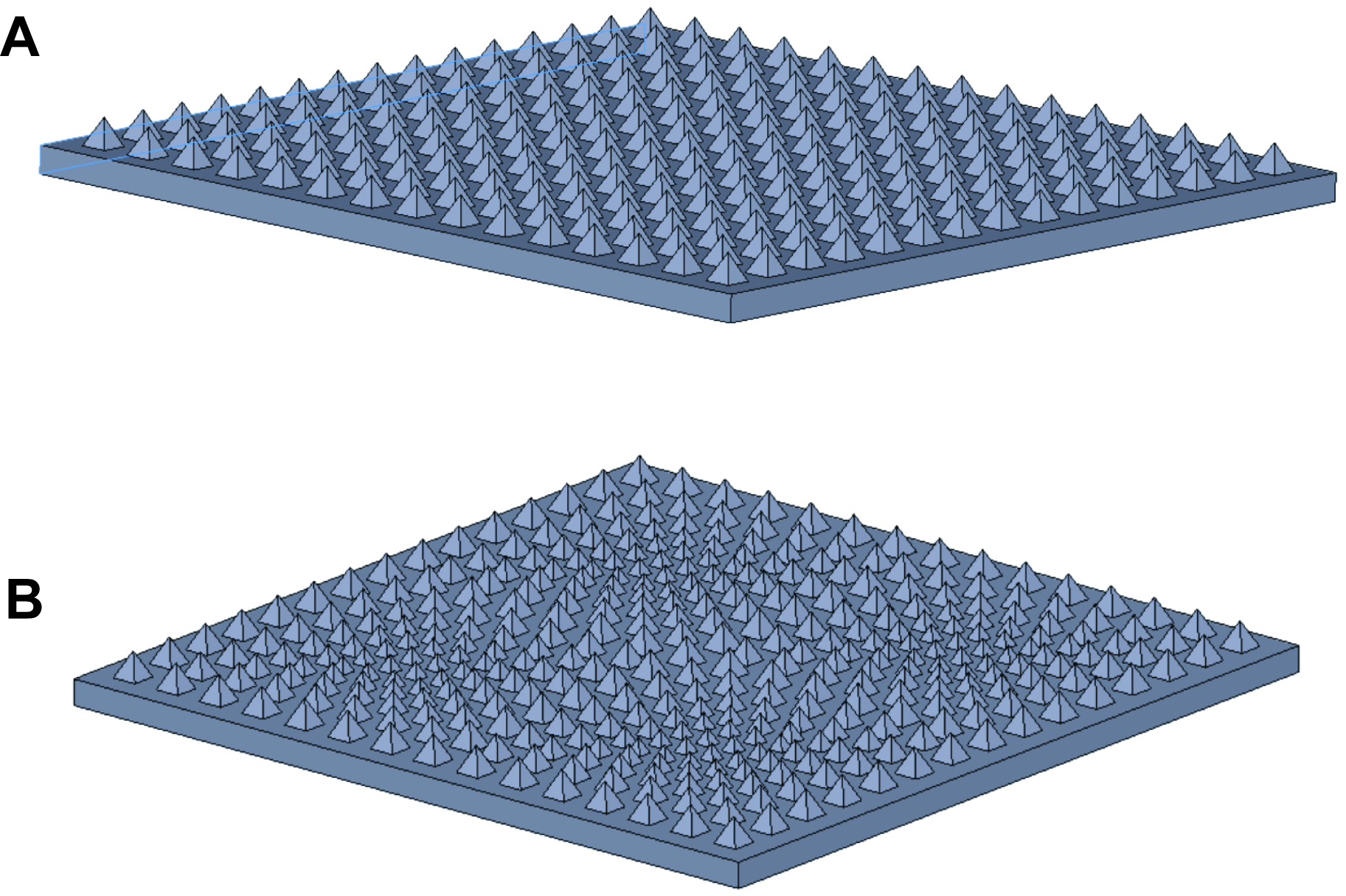


**Figure S25** Theoretical model**s** of (A) homogeneous and (B) hierarchical pyramid microstructures.


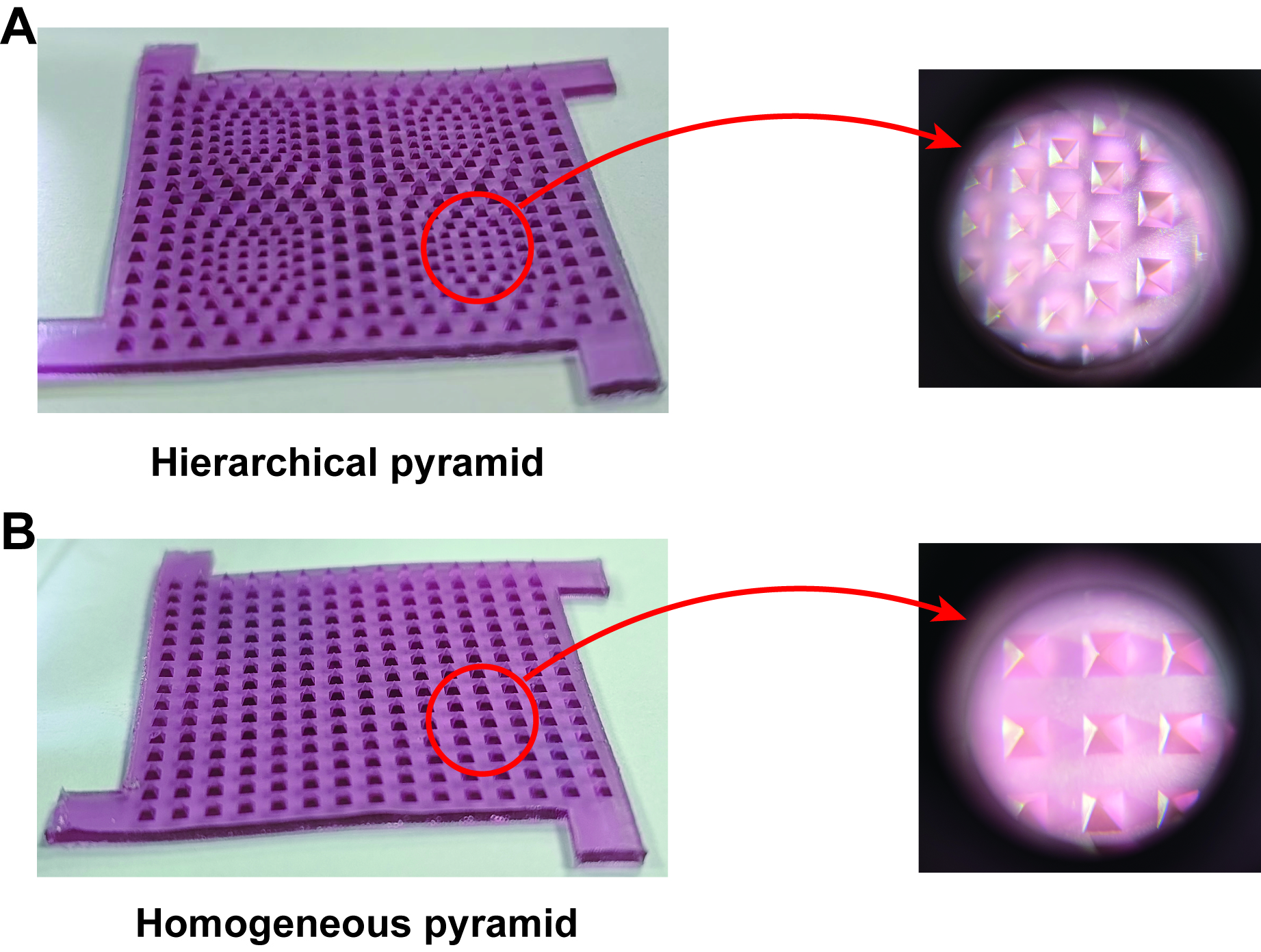


**Figure S26** SAu-PAA/PVP eutectogel with (A) hierarchical pyramid and (B) homogeneous pyramid microstructures. Magnified images of the microstructures were captured using a magnifying microscope lens.


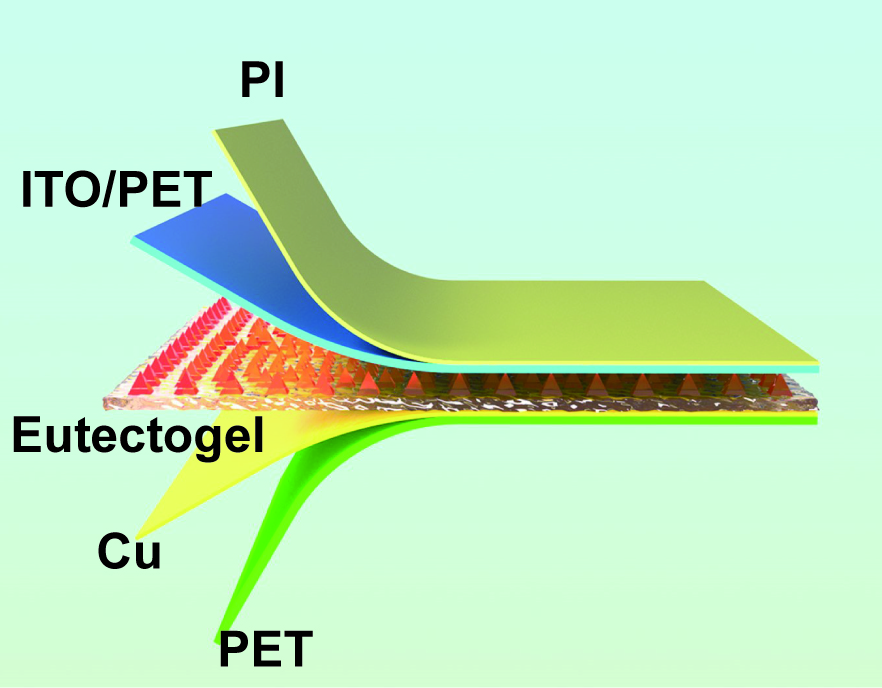


**Figure S27** Structure of the eutectogel-based flexible pressure sensor.


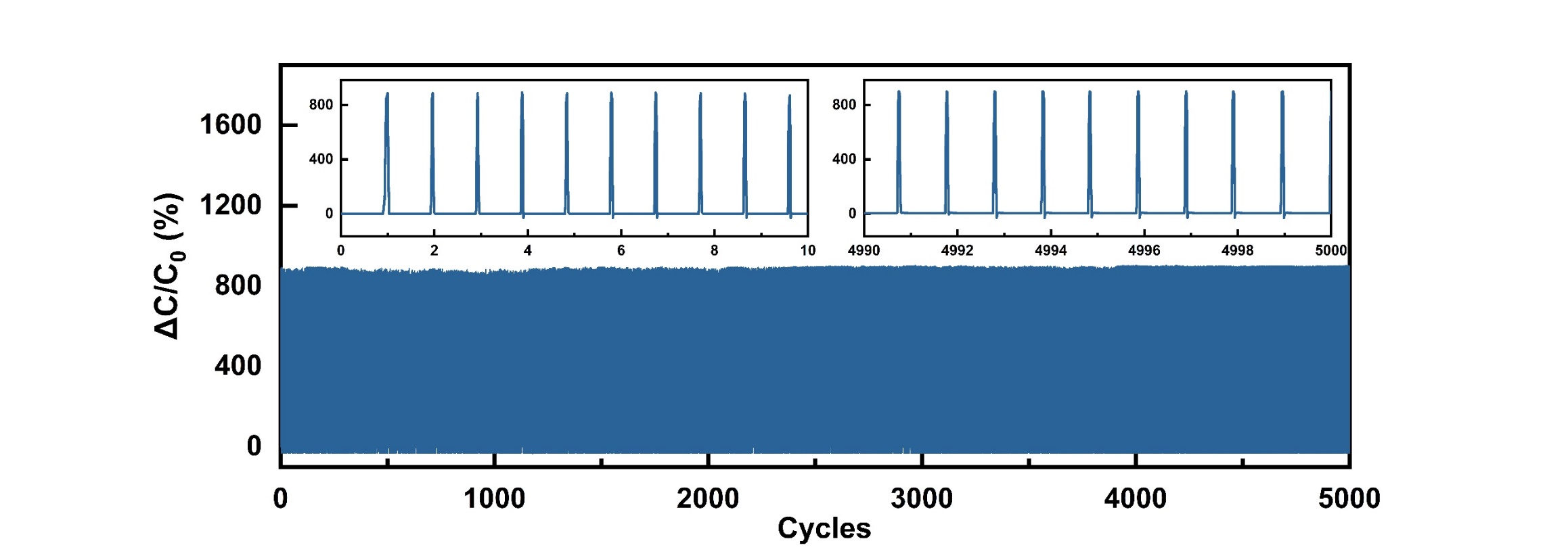


**Figure S28** Relative capacitance changes of the sensor during 5,000 consecutive compression cycles at 0.5 kPa.


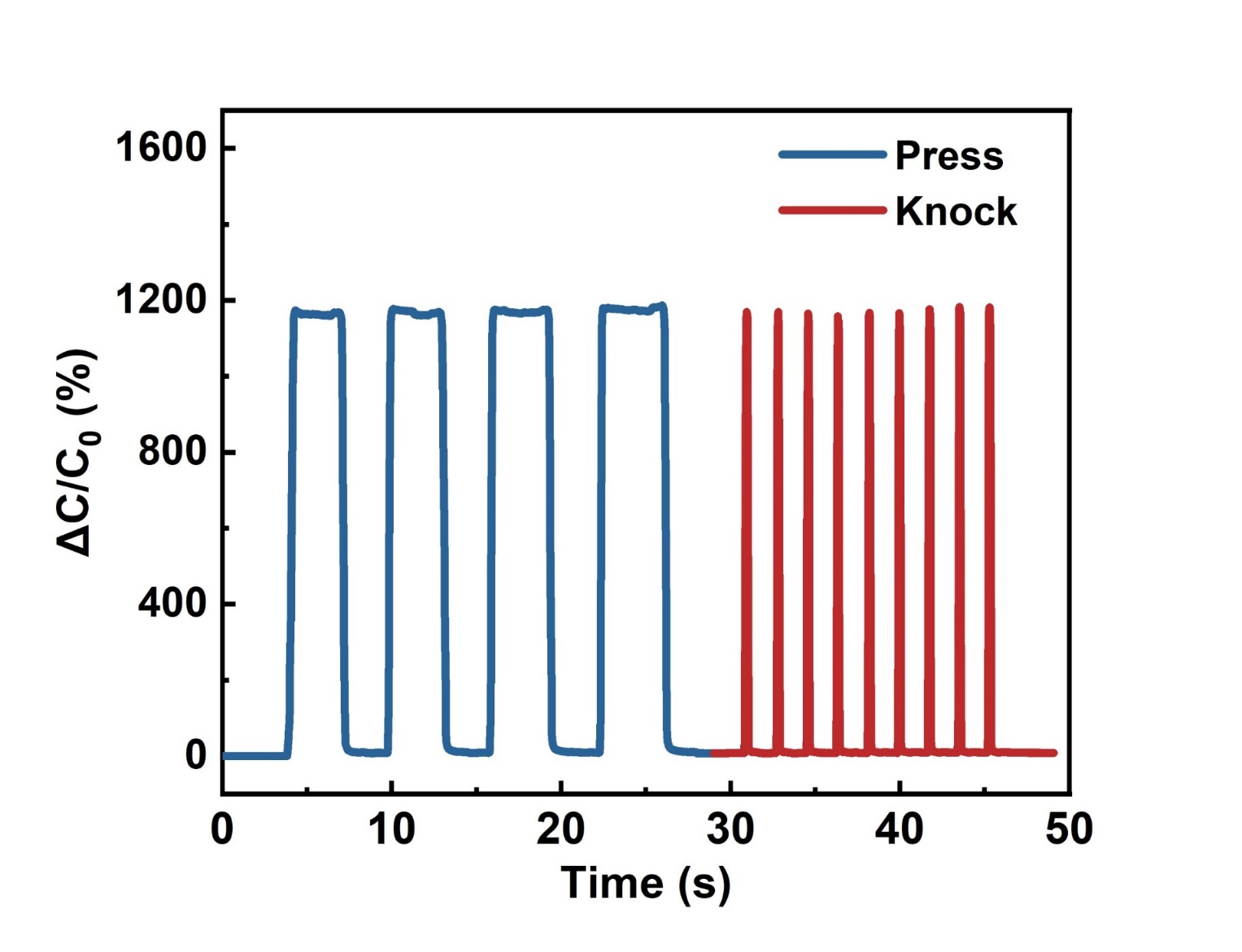


**Figure S29** Capacitance signal changes induced by finger pressing or knocking.


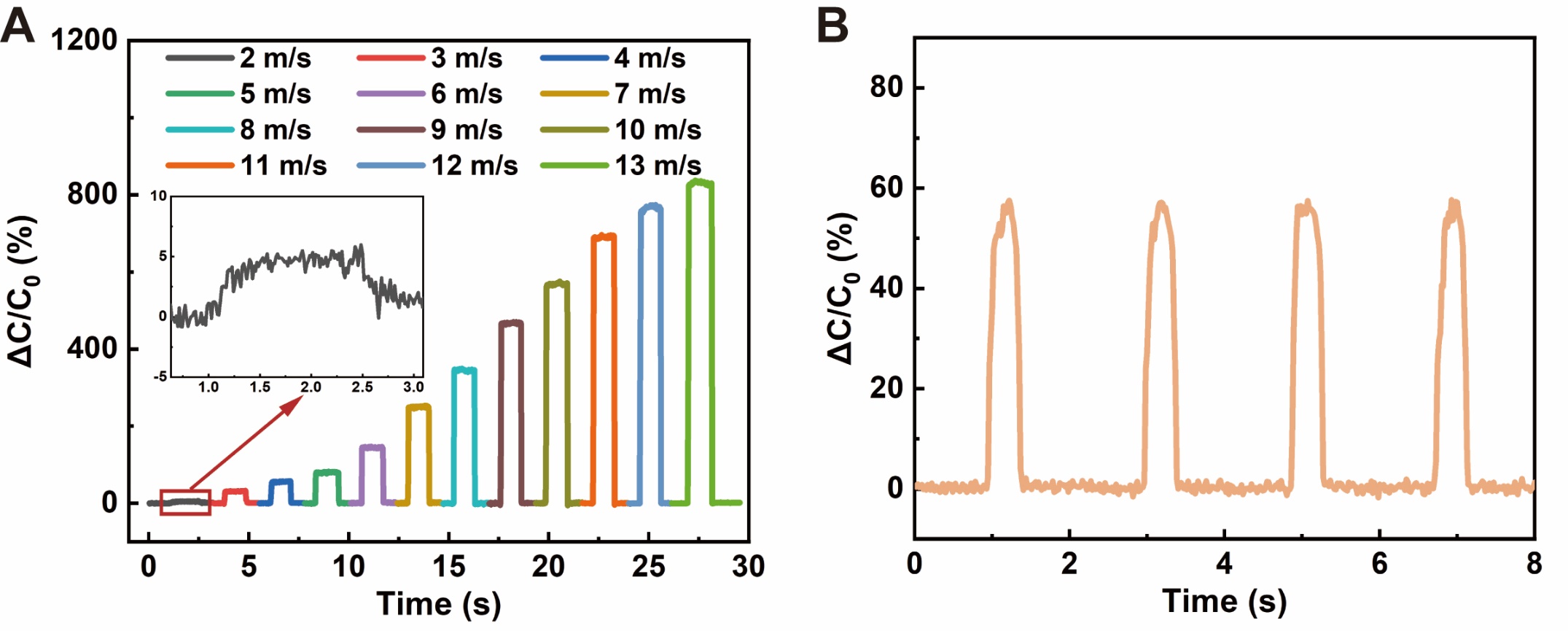


**Figure S30** Capacitance signals of the sensor in response to (A) different wind speeds and (B) blowing air.
